# Association of the Mediterranean Diet, Physical Activity and Related Lifestyle Factors on the Mortality and Major Adverse Cardiovascular Events in Patients with Coronary Heart Disease: A Systematic Review and Meta-Analysis

**DOI:** 10.3390/nu18162589

**Published:** 2026-08-07

**Authors:** Liusheng Mo, Fengyu Qiu, Yueyao Liu, Yuli Zang, Cheng Chen

**Affiliations:** 1 School of Medicine, Hangzhou City University, Hangzhou 310015, China; 2250408004@stu.hzcu.edu.cn (L.M.); 2250408009@stu.hzcu.edu.cn (F.Q.); 2250408003@stu.hzcu.edu.cn (Y.L.); 2 Key Laboratory of Novel Targets and Drug Study for Neural Repair of Zhejiang Province, School of Medicine, Hangzhou City University, Hangzhou 310015, China

**Keywords:** Mediterranean diet, exercise, physical activity, lifestyle, coronary heart disease, all-cause mortality, cardiovascular mortality, major adverse cardiovascular events, secondary prevention, systematic review, meta-analysis

## Abstract

**Introduction:** Evidence linking Mediterranean dietary patterns and physical activity to mortality and major adverse cardiovascular events (MACEs) after coronary heart disease (CHD) diagnosis remains inconclusive. **Methods:** The search covered PubMed, MEDLINE, Scopus, Embase, and CENTRAL from January 2015 through 15 June 2026. Comparable hazard ratios (HRs) were pooled using random-effects models; non-equivalent estimands were reported separately. Risk of bias was evaluated with design-appropriate tools and certainty with GRADE. **Results:** The 40 reports represented 34 data sources and at least 260,354 unique patients, comprising six randomized trials, 30 observational reports, and four comparative nonrandomized studies. In one randomized comparison of Mediterranean and low-fat diets, the HR for MACE was 0.72 (95% CI 0.54 to 0.96; moderate certainty). Observational findings supported inverse associations of closer dietary adherence with all-cause mortality (HR 0.74, 95% CI 0.60 to 0.90; *I*^2^ = 44%) and MACE, whereas cardiovascular mortality remained uncertain. HRs for maintained or continued physical activity relative to persistent inactivity were 0.64 for all-cause mortality (95% CI 0.55 to 0.74; *I*^2^ = 29%) and 0.42 for cardiovascular mortality (95% CI 0.28 to 0.64; *I*^2^ = 72%). MACE findings for diet adherence and habitual physical activity generally favored lower risk. Randomized evidence on exercise or combined dietary and activity programs was sparse. **Conclusions:** Greater Mediterranean diet adherence and habitual physical activity were associated with more favorable CHD outcomes. Standardized, adequately powered trials are needed, especially for exercise and combined strategies.

## 1. Introduction

Coronary heart disease (CHD) remains one of the leading causes of cardiovascular mortality worldwide [1,2,3,4]. According to the World Health Organization (WHO), ischaemic heart disease remains the leading cause of death worldwide, accounting for 13% of all deaths [5]. The clinical burden of CHD is also reflected in major adverse cardiovascular events (MACE), a composite outcome typically including cardiovascular death, nonfatal myocardial infarction, and nonfatal stroke. Furthermore, CHD carries substantial financial costs. Across the countries examined, yearly medical spending per affected individual surpasses average national health expenditure per resident, placing added pressure on individuals, families, and healthcare systems [6,7]. With ongoing population aging, the prevalence of CHD is expected to increase substantially in the coming decades [7,8,9]. Consequently, interventions that deliver both clinical and economic benefits are crucial to mitigating premature mortality and reducing treatment costs associated with CHD.

Current management strategies for CHD primarily rely on pharmacological therapies [10,11,12,13]. However, accumulating evidence indicates that non-pharmacological interventions, particularly dietary modifications and structured physical activity programs, represent essential components of comprehensive CHD management due to their cost-effectiveness and favorable safety profiles [14,15]. Recent clinical guidelines further underscore that sustained adherence to a healthy lifestyle enhances the capacity to manage CHD effectively [16].

Widely recognized for its health benefits, the Mediterranean diet (MD) emphasizes vegetables, fruits, legumes, whole grains, fiber-rich foods, and sources of unsaturated fats, while incorporating moderate amounts of fish and poultry [17]. Higher adherence to MD has been consistently associated with lower cardiovascular risk among adults [18,19,20]. In a randomized controlled trial involving 1002 patients with coronary heart disease, MD intervention reduced the incidence of major cardiovascular events, particularly among male participants, supporting its potential role in secondary cardiovascular prevention [21]. Extra-virgin olive oil (EVOO), the principal fat and a defining food component of the Mediterranean diet [17], may contribute to its cardiovascular benefits through both its monounsaturated fatty acids and phenolic compounds. In human erythrocytes exposed experimentally to hyperglycemic conditions, hydroxytyrosol reduced reactive oxygen species formation, preserved intracellular glutathione, improved calcium homeostasis, and limited phosphatidylserine externalization, a potentially prothrombotic cellular signal [22].

Similarly, regular physical activity (PA), as a core component of non-pharmacological intervention, not only reduces cardiovascular risk in healthy individuals [23,24] but also improves prognosis in patients with existing cardiovascular conditions [25,26]. Research shows that PA enhances myocardial efficiency [27,28], lowers blood pressure [29,30], and improves cardiorespiratory fitness [31,32]. In recreationally active adults, indoor treadmill walking and outdoor hiking produced comparable short-term reductions in systolic and diastolic blood pressure [33]. Repeated aerobic exercise may also improve glucose regulation and lipid metabolism. A preliminary single-participant study in type 1 diabetes reported improved time within the target glucose range and reduced insulin requirements following a multidimensional indoor and outdoor physical activity intervention [34]. In addition, eight weeks of moderate-intensity aerobic training increased high-density lipoprotein cholesterol and reduced glucose, insulin, insulin resistance, triglycerides, total cholesterol, and low-density lipoprotein cholesterol in male smokers and nonsmokers [35]. Beyond these metabolic adaptations, dietary and exercise interventions may share several biological pathways, particularly those related to oxidative stress and inflammation. Exercise-related research suggests that EVOO polyphenols may complement exercise-induced adaptations by attenuating oxidative stress and inflammation, supporting mitochondrial and antioxidant function, and potentially improving aerobic performance and post-exercise recovery [36].

Although adherence to a Mediterranean dietary pattern and regular physical activity are widely recommended for individuals with established coronary heart disease, the available evidence addresses several distinct questions and should not be interpreted uniformly. Studies assigning participants to a Mediterranean diet evaluate the effect of a dietary intervention, whereas observational studies using Mediterranean diet adherence scores evaluate associations between dietary behaviour and clinical outcomes. Similarly, studies of structured physical activity or exercise-based cardiac rehabilitation evaluate the effect of a prescribed programme, whereas studies of physical activity evaluate a self-selected behaviour that may also reflect underlying health status and functional capacity. Interventions or lifestyle indices incorporating both diet and physical activity address a further question concerning the potential value of a combined lifestyle approach; however, such evidence does not necessarily establish independent, additive, or synergistic effects of the two components [37].

Accordingly, this systematic review addressed five separate questions: (1) Among adults with coronary heart disease, how do Mediterranean diet interventions influence mortality and major adverse cardiovascular events? (2) In this population, is adherence to a Mediterranean dietary pattern associated with these outcomes? (3) What is the effect of structured physical activity or exercise-based cardiac rehabilitation? (4) How do mortality and MACE vary according to physical activity among patients with CHD? and (5) What evidence exists for interventions or lifestyle indices jointly incorporating diet and physical activity?

## 2. Methods

This systematic review and meta-analysis followed the Preferred Reporting Items for Systematic Reviews and Meta-Analyses (PRISMA) guideline [38]. The protocol for this study has been registered in the International Prospective Register of Systematic Reviews (PROSPERO, registration code: CRD420251240100).

### 2.1. Search Strategy

A systematic literature search was performed in PubMed, MEDLINE, Scopus, Embase, and the Cochrane Central Register of Controlled Trials (CENTRAL) for records published between 1 January 2015, and 31 December 2025, followed by an updated search up to 15 June 2026. The lower publication-date boundary was prespecified to define a focused synthesis of contemporary evidence and to complement earlier systematic reviews covering overlapping evidence domains. For intervention studies, this boundary was also intended to enhance the relevance of the evidence to contemporary intervention delivery, behaviour-change support, and the evolving clinical context of secondary prevention. Records published in English or Chinese were eligible, with language limits applied through the database interfaces. The complete search strategy is provided in Supplementary Material File S2. A predefined set of keywords was employed to retrieve potentially eligible studies. Furthermore, the reference lists of all included articles were hand-searched, and supplementary searches were conducted on ClinicalTrials.gov. Study authors were contacted for additional data as necessary.

#### 2.1.1. Eligibility Criteria

Study eligibility was defined across five prespecified domains: the target population, interventions or exposures of interest, relevant comparators, outcomes, and eligible research designs, consistent with the PICOS framework.

Population: adults aged ≥18 years with established coronary heart disease (CHD), including previous myocardial infarction or acute coronary syndrome, stable coronary artery disease or angina, ischemic cardiomyopathy, previous percutaneous coronary intervention or coronary artery bypass grafting, or angiographically documented coronary artery disease. Studies enrolling patients with different cardiovascular conditions were considered only if outcome data specific to the CHD subgroup were presented separately or could be isolated from the available information.

Exposure/intervention: eligible interventional studies evaluated a Mediterranean diet intervention, structured exercise, exercise-based cardiac rehabilitation, or a multicomponent lifestyle intervention jointly incorporating diet and physical activity. Eligible observational studies evaluated Mediterranean diet adherence or dietary scores; habitual physical activity; longitudinal changes in physical activity; sedentary behaviour; cardiorespiratory fitness; participation in cardiac rehabilitation; or a lifestyle index jointly incorporating Mediterranean diet and physical activity.

Comparator/reference group: for interventional studies, eligible comparators included usual care, no intervention, or an alternative active intervention. For observational studies, eligible comparisons included a lower Mediterranean diet adherence category, lower habitual physical activity, no favourable increase or a decrease in physical activity, greater sedentary time, lower cardiorespiratory fitness, non-participation in cardiac rehabilitation, or another prespecified reference category.

Outcomes: studies that reported all-cause, cardiovascular mortality, major adverse cardiovascular event (MACE).

Study design: randomized trials, non-randomized comparative intervention studies, prospective or retrospective cohort studies, and case–control studies with an explicit comparator or reference group.

#### 2.1.2. Exclusion Criteria

Studies where participants are severe liver and kidney failure, malignant tumors, cognitive impairment, contraindications to physical activity, as well as pregnant and lactating women, were excluded.

Non-original studies and studies with other methodological designs (e.g., reviews, comments, letters, and editorials, etc.).

Prospective cohort studies were excluded if they did not compare defined exposure groups or report associations for a continuous exposure measure.

#### 2.1.3. Definition and Classification of Composite Cardiovascular Outcomes

MACE was not considered a uniform outcome across studies. Before quantitative synthesis, the components of every study-defined composite cardiovascular outcome were extracted independently by two reviewers (M.L.S. and Q.F.Y.) and classified according to a predefined clinical hierarchy.

Core MACE comprised a composite of at least two major cardiovascular outcomes among cardiovascular death, non-fatal myocardial infarction, and non-fatal stroke.

Expanded MACE/MACCE comprised core MACE components together with one or more additional major cardiovascular outcomes, such as recurrent acute coronary syndrome or unstable angina, urgent or unplanned coronary revascularization, hospitalization for heart failure, or hospitalization for another acute cardiovascular condition.

Recurrent coronary-event composites included combinations of coronary death, myocardial infarction, recurrent acute coronary syndrome, unstable angina, or coronary revascularization but did not necessarily include stroke.

Other composite cardiovascular outcomes were those containing substantially different outcomes, such as new atrial fibrillation, elective procedures, symptom-driven medical visits, all-cause hospitalization, or non-cardiovascular events.

MACCE was not assumed to be equivalent to MACE solely on the basis of its label. It was classified according to its reported components. Stroke, recurrent acute coronary syndrome, revascularization, and hospitalization reported as individual outcomes were not treated as MACE. Revascularization and hospitalization were accepted as components of an expanded composite only when they were urgent, unplanned, or clearly attributable to an acute cardiovascular condition. Studies with clinically incompatible composite definitions were not pooled in the same primary analysis.

### 2.2. Study Selection

Records identified through the database searches were imported into EndNote 2025.1 (Build 19456; Clarivate, Philadelphia, PA, USA) and Rayyan (Rayyan Systems Inc., Cambridge, MA, USA), an online platform designed to facilitate the screening of large record sets. Any AI-assisted relevance suggestions provided by Rayyan were treated as non-binding and were not used to automatically include or exclude records or to apply an AI-based stopping rule. Data extraction was performed in Microsoft Excel. Two reviewers (M.L.S. and Q.F.Y.) independently evaluated titles and abstracts before examining the full texts of potentially eligible articles. Differences at either stage, including those concerning the reasons for full-text exclusion, were discussed with the third reviewer (C.C.), until consensus was reached.

### 2.3. Data Extraction and Risk of Bias Assessment

Two reviewers (M.L.S. and Q.F.Y.) independently collected data from each eligible study using a prespecified standardized form to ensure consistency and reduce extraction bias. Any disagreements were settled by consensus or, when required, by involving a third reviewer. The information recorded comprised publication details (first author, year, and study location), study design, participant characteristics (sample size, sex, and mean age), exposure or intervention characteristics (definition, assessment instrument or intervention protocol, and comparator or reference group), and outcome data (outcome definition, number of events, and length of follow-up). All extracted information was compiled in a Microsoft Excel spreadsheet. When essential information was missing or insufficiently reported, clarification was sought from the corresponding authors through at least two contact attempts.

Reports were linked to their parent cohorts using the trial or registry name, country, recruitment period, eligibility criteria, sample size, participating centres, and author information. Two reviewers (M.L.S. and Q.F.Y.) independently assigned each report a parent-cohort identifier, any differences in coding were reconciled through discussion, with C.C. consulted as a third reviewer when required. Reports based on identical, nested, or potentially overlapping source populations were flagged before quantitative synthesis.

Bias judgments were made separately for each result included in a synthesis, with the assessment approach determined by the relevant intervention or exposure contrast rather than the overall design classification of the source study. Results from randomized comparisons were evaluated using Cochrane’s RoB 2 tool, focusing on the effect of assignment to the intervention. Comparative non-randomized intervention effects were assessed using the 20 November 2025 draft version of ROBINS-I V2 across six domains: bias due to confounding, bias arising from classification of intervention, bias in selection into the study, bias due to missing data, bias in measurement of the outcome, and bias in selection of the reported result. Observational associations involving Mediterranean diet adherence, physical activity, sedentary behaviour, or cardiorespiratory fitness were assessed using ROBINS-E across seven domains: bias due to confounding, bias arising from measurement of the exposure, bias in selection of participants into the study or analysis, bias due to post-exposure interventions, bias due to missing data, bias arising from measurement of the outcome, and bias in selection of the reported result.

Two reviewers (M.L.S. and Q.F.Y.) independently appraised the risk of bias for every included study. Discrepant ratings were discussed until consensus was reached, and any unresolved cases were adjudicated by a third reviewer (C.C.).

### 2.4. Certainty of the Evidence

For every body of evidence, two reviewers (M.L.S. and Q.F.Y.) separately assigned a certainty level using the Grading of Recommendations Assessment, Development and Evaluation (GRADE) approach. They discussed any differences in their initial ratings, and cases that remained unresolved were referred to a third reviewer (C.C.) for adjudication. Consistent with the prespecified review questions, GRADE assessments were conducted separately for each outcome (all-cause mortality, cardiovascular mortality, and MACE), exposure or intervention domain (Mediterranean diet intervention, Mediterranean diet adherence, structured exercise or exercise-based cardiac rehabilitation, physical activity, and combined diet-and-physical-activity interventions or lifestyle indices), and study design (randomized versus non-randomized or observational evidence).

Under the conventional GRADE framework used in this review, evidence from randomized controlled trials started at high certainty, whereas observational evidence started at low certainty. The RoB 2, ROBINS-I V2 and ROBINS-E assessments informed the GRADE risk-of-bias domain. Certainty was downgraded by one level for serious concerns or by two levels for very serious concerns regarding risk of bias, inconsistency, indirectness, or imprecision, and by one level when publication bias was strongly suspected. After evaluating all potential reasons for downgrading, observational evidence was considered for upgrading when the association was large or very large, a dose–response gradient was present, or all plausible residual confounding would be expected to weaken the observed association. Overall certainty was then classified as high, moderate, low, or very low. Detailed explanations for all domain-level judgments are provided in Supplementary Material File S1: Table S1.

### 2.5. Statistical Analysis

All quantitative syntheses were performed using Review Manager (RevMan) software, version 5.4 (The Cochrane Collaboration, London, UK). Given that all-cause mortality, cardiovascular mortality, and MACE were time-to-event outcomes, with considerable variation in follow-up durations among studies, hazard ratios (HRs) with corresponding 95% confidence intervals (CIs) were used as the primary summary effect measure for these outcomes. HRs were not treated as approximations of risk ratios (RRs), and effect estimates based solely on cumulative event counts, such as RRs or odds ratios (ORs), were not combined with HR-based estimates for mortality or MACE outcomes.

For each eligible study, the natural logarithm of the HR [log(HR)] and its standard error (SE) were extracted or calculated for meta-analysis. When log(HR) and SE were not directly reported, log(HR) was calculated as ln(HR), and the SE was derived from the reported 95% CI using the formula: SE = [ln(upper CI) − ln(lower CI)]/3.92. The generic inverse-variance method was used to pool log(HR) estimates. Pooled estimates were subsequently exponentiated and presented as HRs with 95% CIs. An HR < 1 indicated a lower hazard of the outcome in participants with higher Mediterranean diet adherence, higher physical activity or physical activity exposure, or combined Mediterranean diet and physical activity intervention, compared with the corresponding reference group.

When multiple effect estimates were reported in the same study, the most fully adjusted HR was preferentially extracted to minimize confounding. For randomized controlled trials, intention-to-treat estimates were prioritized when available. If a study reported several exposure contrasts, the estimate corresponding to the predefined comparison of interest was used, including high versus low Mediterranean diet adherence, higher versus lower physical activity or exercise exposure, or intervention versus control. Estimates based on different exposure scales, such as categorical comparisons and continuous score increments, were not assumed to be directly equivalent and were explored using subgroup or sensitivity analyses where appropriate.

Effect estimates were classified according to both study design and the causal question addressed before quantitative synthesis. Randomized trials estimating the effect of assignment to a lifestyle intervention were not pooled with observational studies estimating adjusted associations with lifestyle exposures, even when both reported hazard ratios. A common effect measure was therefore considered necessary but not sufficient for quantitative synthesis.

Randomized intervention effects, comparative non-randomized intervention effects, and observational exposure associations were synthesized separately. Within observational evidence, pooling was undertaken only when the exposure definition, contrast, outcome definition, and effect scale were considered sufficiently comparable. Estimates based on high-versus-low exposure categories were not pooled with estimates expressed per unit increase in a lifestyle score unless a common scale could be established. Multicomponent lifestyle indices incorporating diet, physical activity, rest, or social factors were not pooled with estimates of Mediterranean diet adherence alone.

Because clinical and methodological heterogeneity was anticipated across studies, including differences in study design, participant characteristics, intervention or exposure definitions, outcome definitions, and follow-up duration, random-effects models were prespecified as the primary analytical approach. Heterogeneity across studies was examined using Cochran’s Q test and quantified using the *I*^2^ statistic. *I*^2^ values of approximately 25%, 50%, and 75% were interpreted as indicating low, moderate, and high heterogeneity, respectively. Potential sources of heterogeneity were explored through prespecified subgroup analyses according to intervention type, disease category, exposure definition, and study design where sufficient data were available.

Sensitivity analyses were performed to examine the stability of the pooled estimates, including leave-one-out analyses and analyses excluding studies with distinct exposure definitions, atypical outcome definitions, or substantial methodological differences. Funnel plots and Egger’s regression test were planned when at least 10 independent studies contributed to the same outcome, evidence stratum, and estimand-specific meta-analysis. Reports derived from the same parent cohort were not counted as independent studies for this purpose. Continuous outcomes, such as low-density lipoprotein cholesterol, were summarized as mean differences (MDs) when measured on the same scale, or standardized mean differences (SMDs) when different measurement scales were used.

## 3. Results

**Figure 1:**
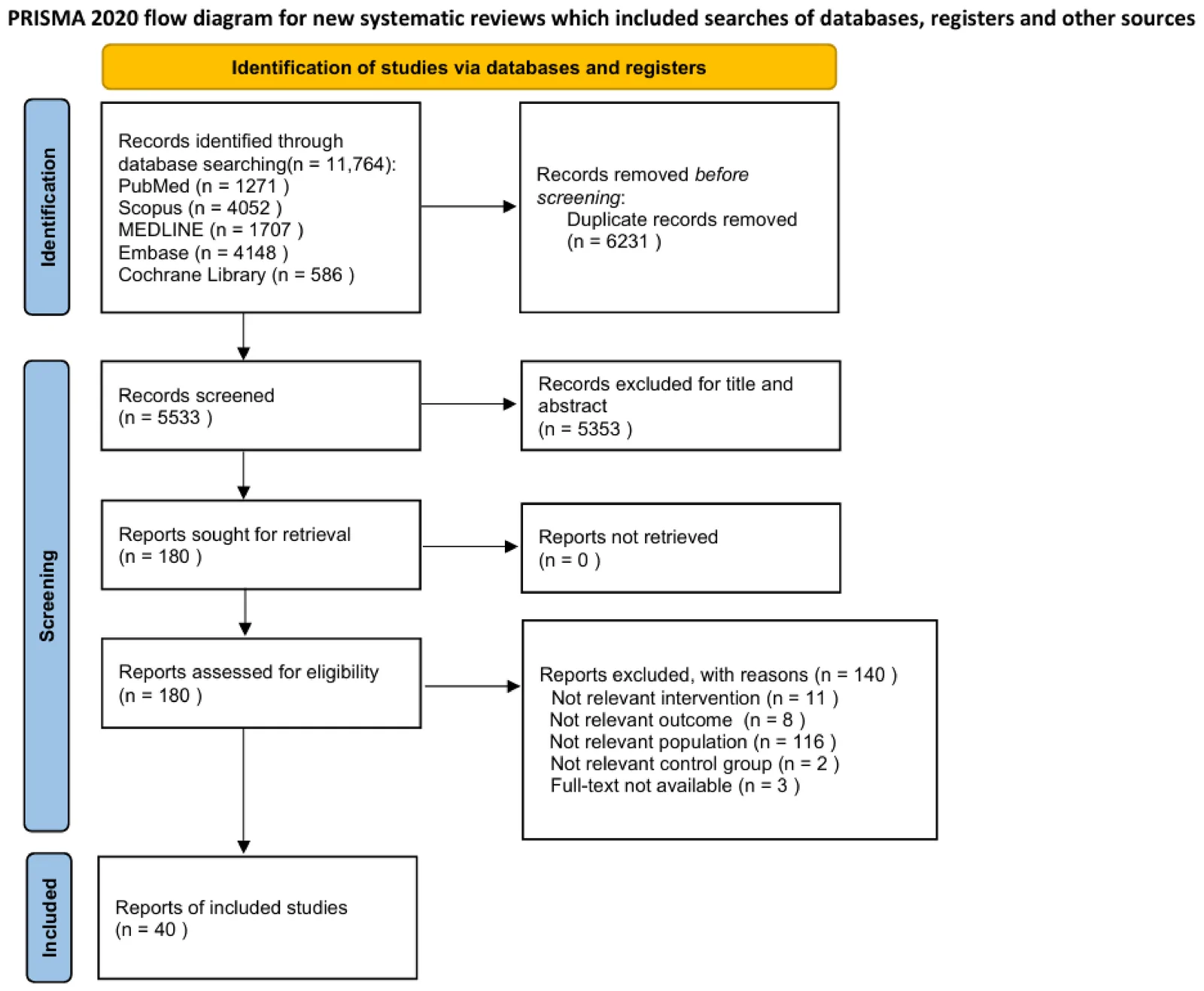
PRISMA flow diagram for study selection.

The PRISMA flow diagram summarizes the study selection process for this systematic review and meta-analysis (Figure 1). A total of 11,764 records were identified through database searches. After removing duplicate records (*n* = 6231), titles and abstracts were screened, and 180 full-text articles were retrieved for detailed assessment. Ultimately, 40 eligible reports representing 34 parent cohorts or data sources were included. Five groups of reports originated from identical, nested, or potentially overlapping source populations: STABILITY, the Korean NHIS ACS cohort, the Australian 45 and Up Study, SWEDEHEART, and NHANES. After counting exact duplicates and subsets once and applying a conservative rule to the partially overlapping registry samples, the review represented at least 260,354 non-duplicated participants (Supplementary Material File S1: Table S3). Among the 40 included reports, six evaluated randomized intervention effects, four reports evaluated comparative non-randomized interventions, whereas 30 evaluated observational lifestyle exposures. With respect to exposure type, 10 reports evaluated the Mediterranean diet, 30 examined exercise or physical activity, and five assessed combined Mediterranean diet and exercise-related lifestyle patterns. Where sufficient and methodologically comparable data were available, these studies were further included in the meta-analysis. Studies that did not meet these requirements were presented individually or synthesized narratively, with the study-specific decision documented in Supplementary Material File S1: Table S4.

### 3.1. Study Characteristics

The characteristics of the studies evaluating the Mediterranean diet are presented in Table 1. The eligible studies were published between 2015 and 2026. Geographically, three studies were conducted in the United States [39,40,41] and two in Spain [21,42]. In addition, one study was conducted in Italy [43], one in Greece [44], and one in Hong Kong, China [45]; one study was a multi-regional European investigation involving Italy, Scotland, and other European regions [46]; and one was a global multicenter study including participants from 39 countries [47]. In most studies, dietary exposure was assessed using a Mediterranean diet score. Two studies evaluated Mediterranean diet adherence, one used the alternate Mediterranean Diet score, and one assessed dietary exposure based on dietary intervention records. The timing of dietary assessment varied across studies. Dietary exposure was generally assessed once at study entry among patients with established CHD or at the index coronary presentation. No study assessed lifetime Mediterranean diet adherence. Further details are provided in Supplementary Material File S1: Table S5.

Table 2 summarizes the characteristics of the 30 eligible studies evaluating exercise or physical activity exposure. Across the eligible studies, publication dates ranged from 2016 through 2025. In terms of geographical distribution, four studies were conducted in South Korea [48,49,50,51], while three studies each were conducted in China [52,53,54], Australia [55,56,57], and the United States [58,59,60]. Two studies each were conducted in the United Kingdom [61,62], France [63,64], and Sweden [65,66]. In addition, one study each was conducted in Chile [67], Spain [68], Turkey [69], Brazil [70], Japan [71], Taiwan, China [72], Finland [73], and Norway [74]. One study was jointly conducted in Italy and Spain [75], and two were multinational studies, involving participants from 39 [76] and 45 [77] countries, respectively.

Five studies [42,43,44,47,68] published between 2016 and 2026 also evaluated lifestyle patterns combining the Mediterranean diet and physical activity among patients with CHD. Of these, two were conducted in Spain [42,68], one each in Italy [43] and Greece [44], and one was a multinational study involving participants from 39 countries [47].

Given the variability in the definition of composite endpoints across different studies, the specific components and their classifications are elaborated in the Supplementary Material File S1: Table S2.

### 3.2. Association Between the Mediterranean Diet and All-Cause Mortality

**Figure 2:**
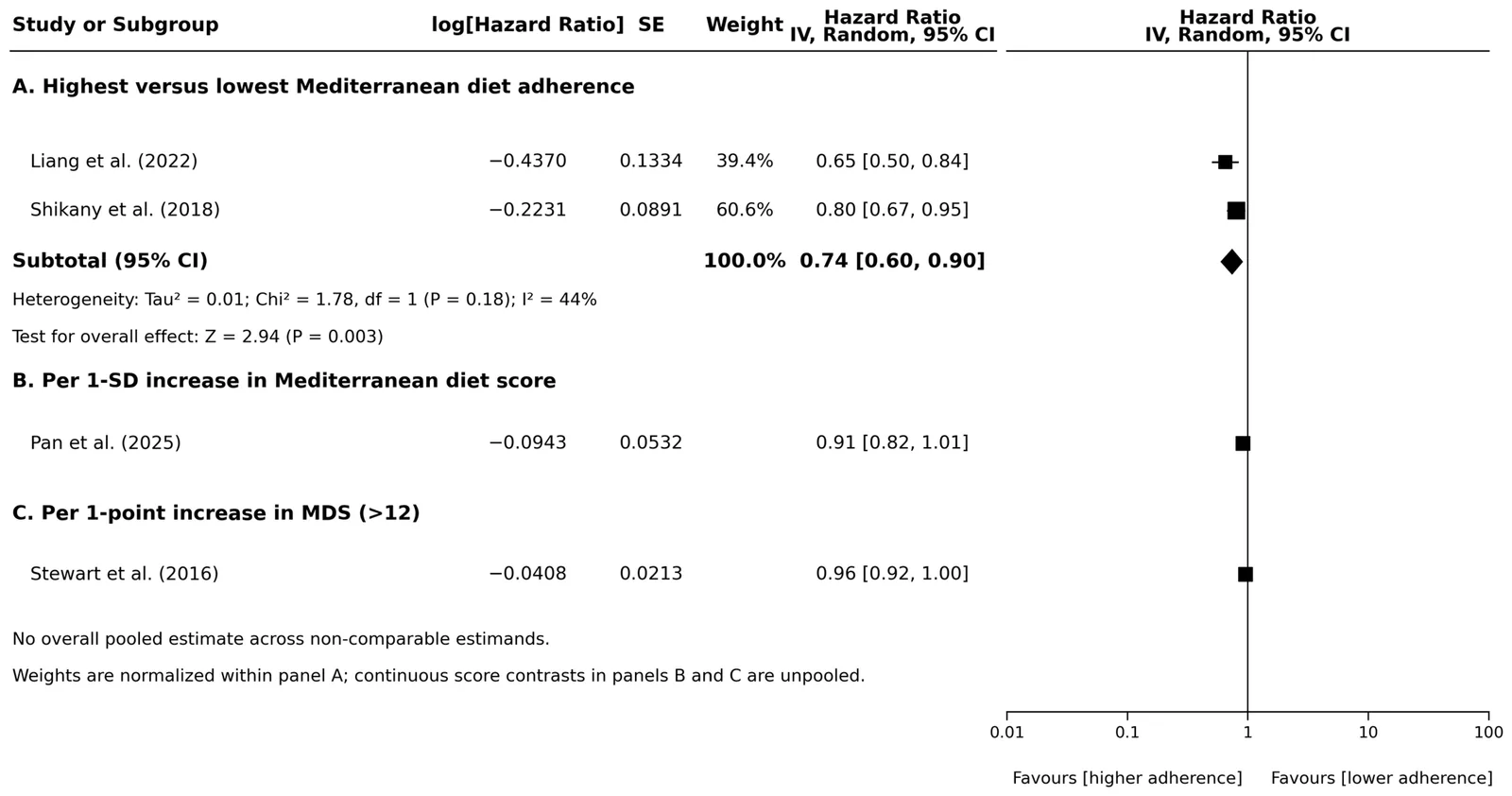
Forest display of adjusted hazard ratios for observational Mediterranean diet adherence and all-cause mortality [39,40,41,76].

We first evaluated the relationship between Mediterranean diet adherence and all-cause mortality in patients with coronary heart disease. Four studies [39,40,41,47] reported data on the relationship between Mediterranean diet adherence and all-cause mortality in this population. Among studies using a highest-versus-lowest Mediterranean diet adherence contrast, higher adherence was associated with lower all-cause mortality (pooled HR = 0.74, 95% CI: 0.60 to 0.90; *I*^2^ = 44%). Pan et al. reported an HR of 0.91 (95% CI: 0.82 to 1.01) per 1-SD increase in Mediterranean diet score, whereas Stewart et al. reported an HR of 0.96 (95% CI: 0.92 to 1.00) per one-point increase among participants with an MDS >12. Because these estimates represented different score scales and contrasts, these findings were presented separately rather than pooled in the categorical adherence meta-analysis shown in Figure 2.

### 3.3. Association Between the Mediterranean Diet and Cardiovascular Mortality

**Figure 3:**
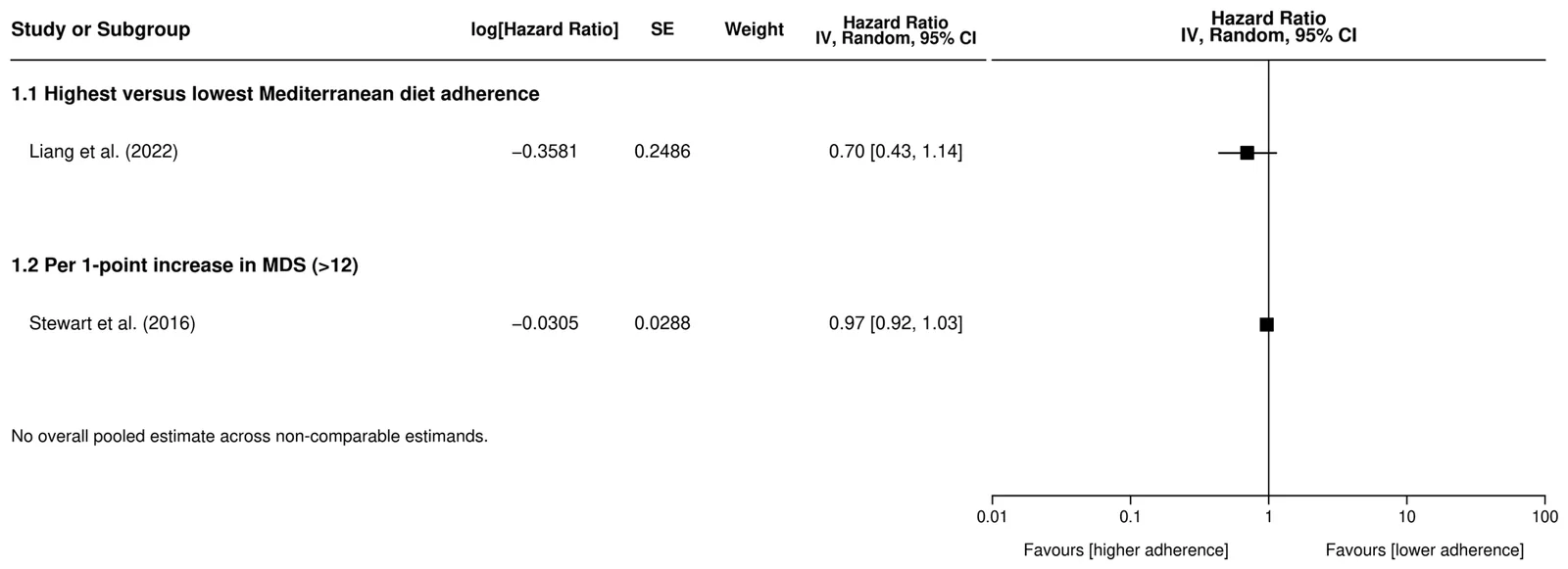
Forest plot of hazard ratios for the association between the Mediterranean diet and cardiovascular mortality in patients with coronary heart disease [40,47].

We then examined the association between the Mediterranean diet and cardiovascular mortality among patients with CHD. Given the limited number of available studies, only two studies [40,47] were included in this analysis. The two available studies used non-equivalent exposure contrasts. Liang et al. reported an HR of 0.70 (95% CI: 0.43 to 1.14) for the highest versus lowest adherence category, whereas Stewart et al. reported an HR of 0.97 (95% CI: 0.92 to 1.03) per one-point increase in MDS among participants with an MDS >12. These estimates were therefore presented separately without a pooled effect estimate (Figure 3).

### 3.4. Mediterranean Diet Intervention, Adherence, and Joint Lifestyle Indices in Relation to MACE

Five reports [21,39,42,43,47] provided hazard ratios for MACE but addressed three distinct research questions and were therefore not combined in a common meta-analysis. In the CORDIOPREV randomized trial, assignment to a Mediterranean diet rather than a low-fat diet was associated with a lower hazard of MACE (HR = 0.72, 95% CI 0.54 to 0.96).

Three observational studies evaluated Mediterranean diet adherence. Cangemi et al. reported an adjusted HR of 0.49 (95% CI 0.29 to 0.82) and Shikany et al. reported an adjusted HR of 0.78 (95% CI 0.62 to 0.98) for higher versus lower adherence. Stewart et al. reported an HR of 0.95 (95% CI 0.92 to 0.99) per one-point increase in the Mediterranean diet score. Because the Stewart estimate used a continuous score increment whereas the other studies used categorical adherence contrasts, these estimates were displayed individually and were not pooled.

**Figure 4:**
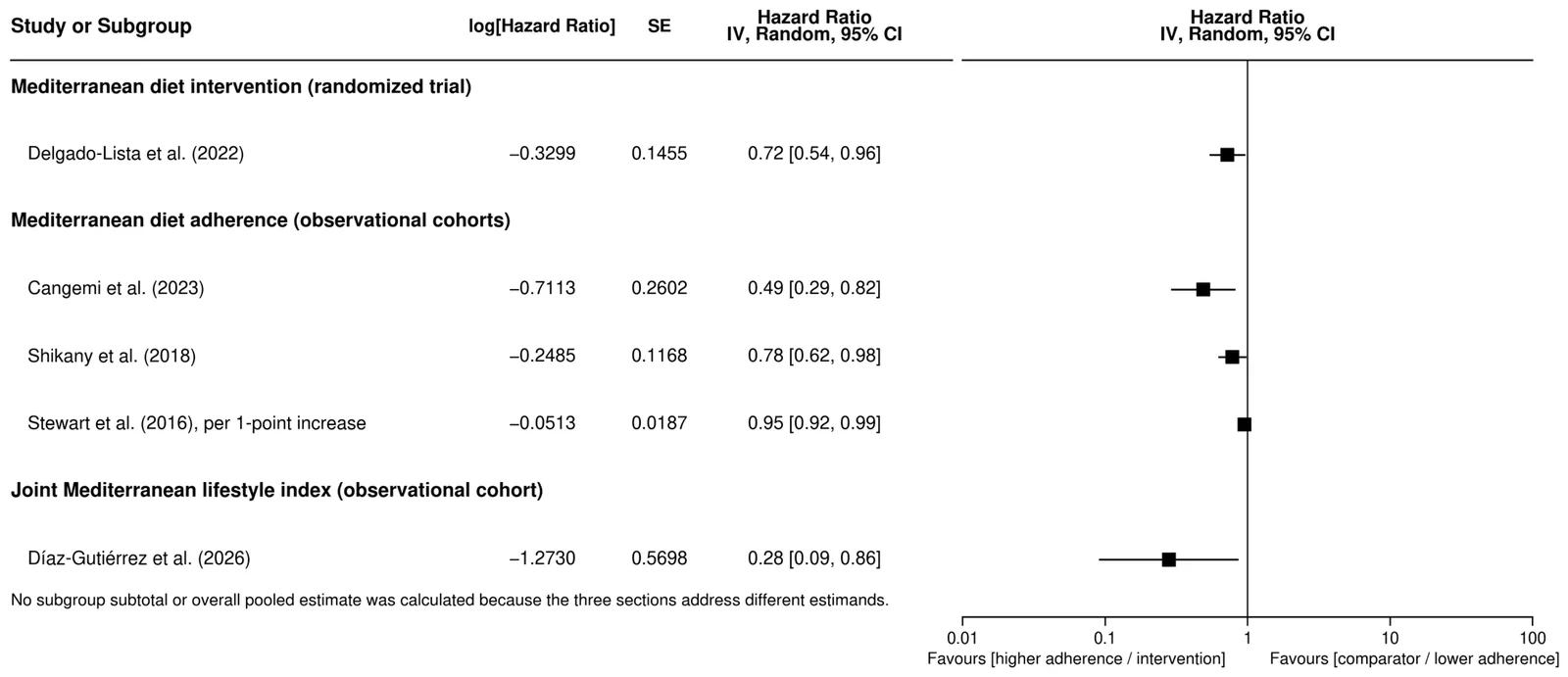
Forest display of the randomized intervention effect and adjusted observational associations between Mediterranean diet-related exposures and major adverse cardiovascular events [21,39,42,43,47].

Díaz-Gutiérrez et al. evaluated the MEDLIFE index, which jointly incorporates Mediterranean diet, physical activity, rest, and social habits. For adverse cardiovascular events, the HR comparing greater with lower adherence to the combined lifestyle pattern was 0.28 (95% CI 0.09 to 0.86). Because this exposure was not a dietary intervention or a measure of dietary adherence alone, it was treated as a separate joint-lifestyle question. Figure 4 displays the three evidence categories separately, without an overall pooled estimate.

### 3.5. Association of Habitual Physical Activity with All-Cause Mortality

All-cause mortality data according to physical activity were available from 11 studies [49,51,54,57,58,59,62,64,65,74,76] of patients with CHD. Because the studies used different physical-activity contrasts, analyses were stratified by estimand. The pooled HR comparing maintained or continued activity with persistent inactivity was 0.64 (95% CI 0.55 to 0.74; *I*^2^ = 29%; *p* < 0.00001). Meeting a recommended physical-activity target, compared with inactivity, was also associated with lower mortality (HR = 0.69, 95% CI: 0.53 to 0.90; *I*^2^ = 90%; *p* = 0.006). For study-defined higher versus lower categorical physical-activity levels, the pooled HR was 0.61 (95% CI: 0.47 to 0.79; *I*^2^ = 80%; *p* = 0.0002).

**Figure 5:**
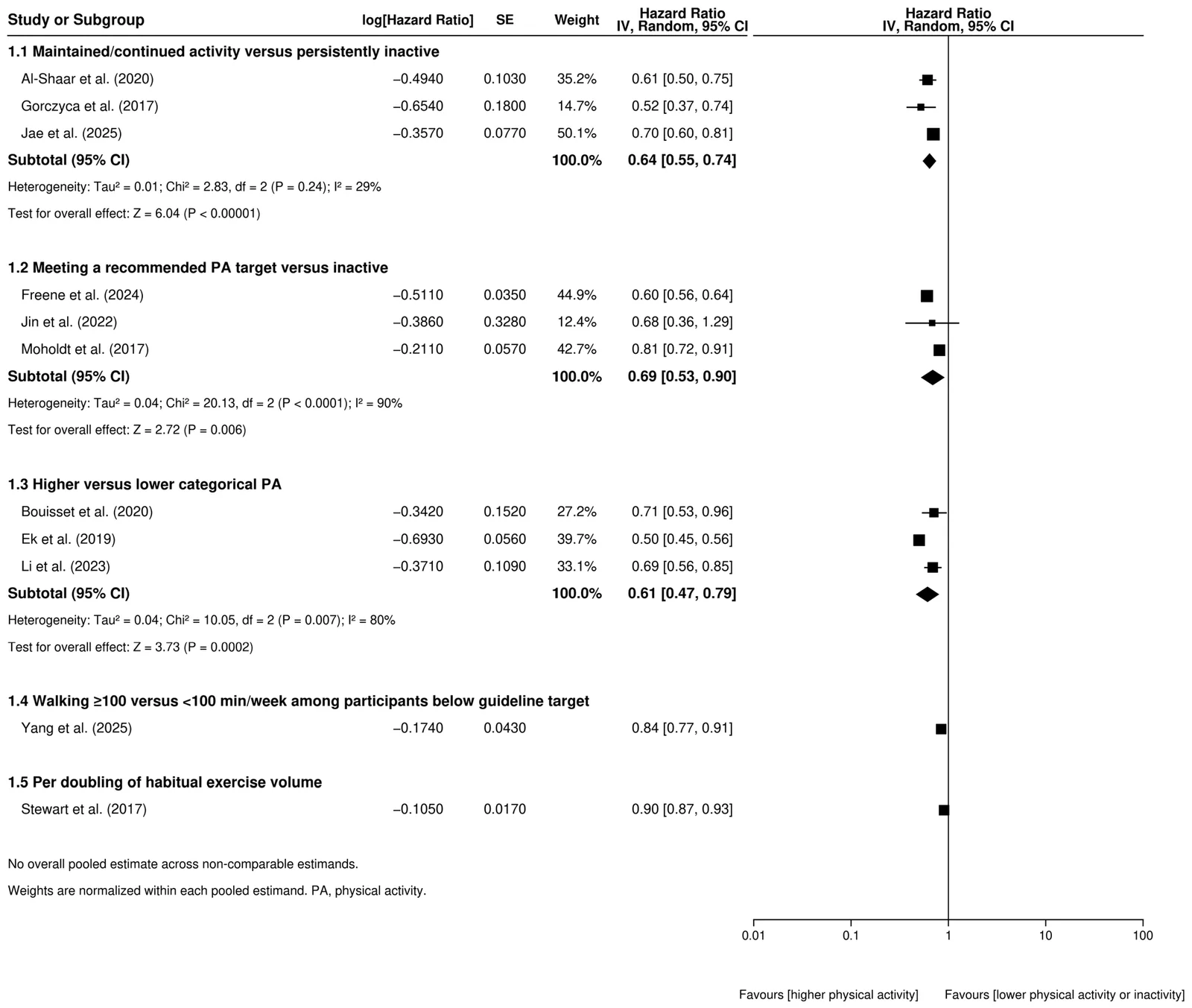
Forest plot of hazard ratios for the association of habitual physical activity with all-cause mortality in patients with CHD [47,49,51,54,57,58,59,62,64,65,74].

Two additional estimates represented distinct contrasts and were not pooled with these subgroups. Among participants not meeting overall physical-activity recommendations, walking for at least 100 min/week was associated with lower mortality than walking for less than 100 min/week (HR = 0.84, 95% CI: 0.77 to 0.91). Each doubling of physical activity volume was associated with a lower mortality risk (HR = 0.90, 95% CI: 0.87 to 0.93). No overall estimate was calculated across these non-comparable estimands (Figure 5).

### 3.6. Association of Habitual Physical Activity with Cardiovascular Mortality

Eight studies [49,54,57,58,59,73,74,76] examined the association between physical activity and cardiovascular mortality. Analyses were stratified according to the physical-activity contrast. Maintaining or continuing physical activity, compared with remaining persistently inactive, was associated with lower cardiovascular mortality (HR = 0.42, 95% CI: 0.28 to 0.64; *I*^2^ = 72%; *p* < 0.0001). Meeting a recommended physical-activity target, compared with inactivity, was also associated with lower cardiovascular mortality (HR = 0.67, 95% CI: 0.47 to 0.95; *I*^2^ = 91%; *p* = 0.02).

**Figure 6:**
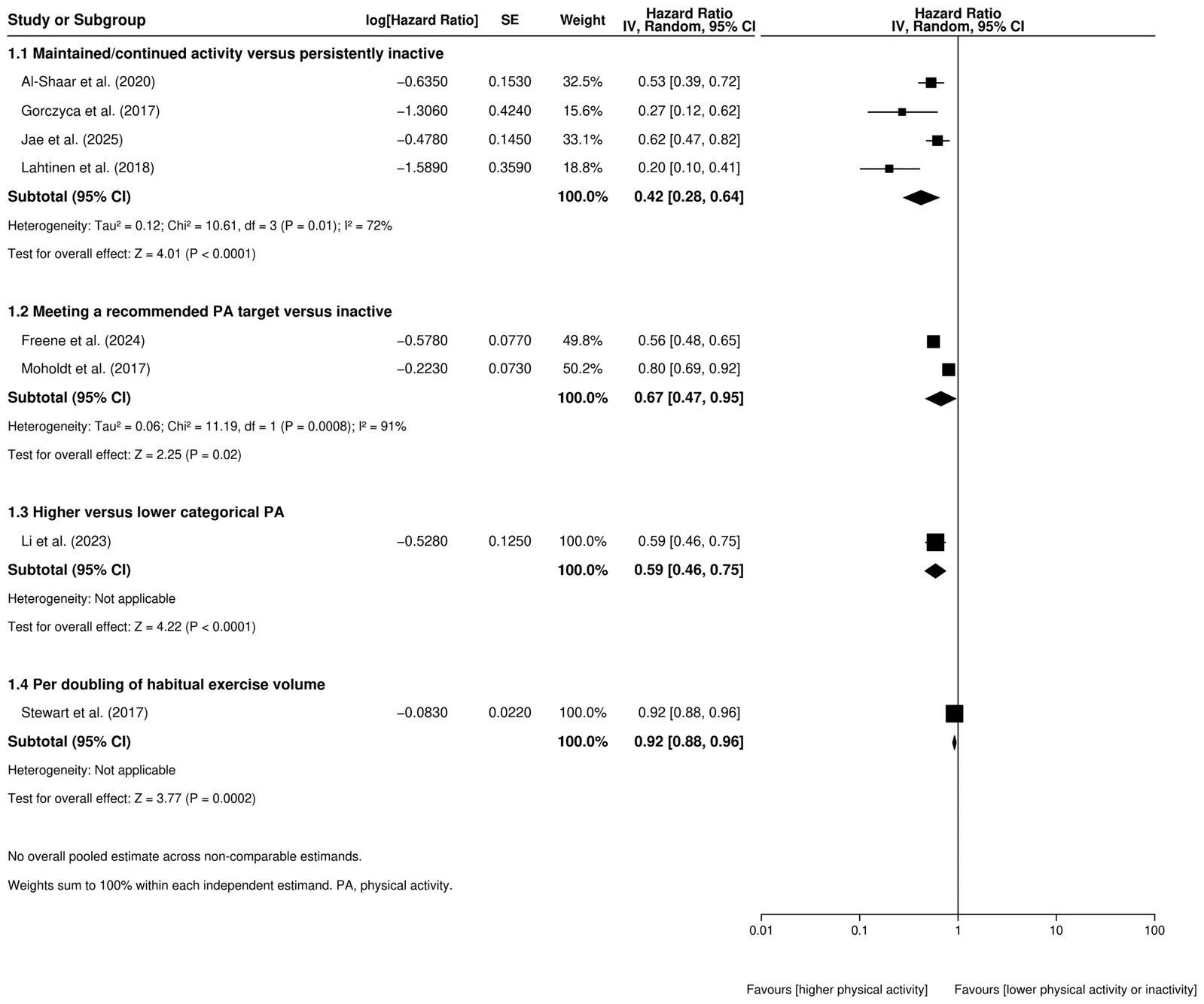
Forest plot of hazard ratios for the association of habitual physical activity with cardiovascular mortality in patients with coronary heart disease [49,54,57,58,59,73,74,76].

In the single study comparing higher with lower categorical physical activity, the HR was 0.59 (95% CI: 0.46 to 0.75; *p* < 0.0001). Another study reported an HR of 0.92 (95% CI: 0.88 to 0.96; *p* = 0.0002) per doubling of physical activity volume. Because these estimates represented different estimands, no overall pooled estimate was calculated (Figure 6).

### 3.7. Association of Habitual Physical Activity with MACE

**Figure 7:**
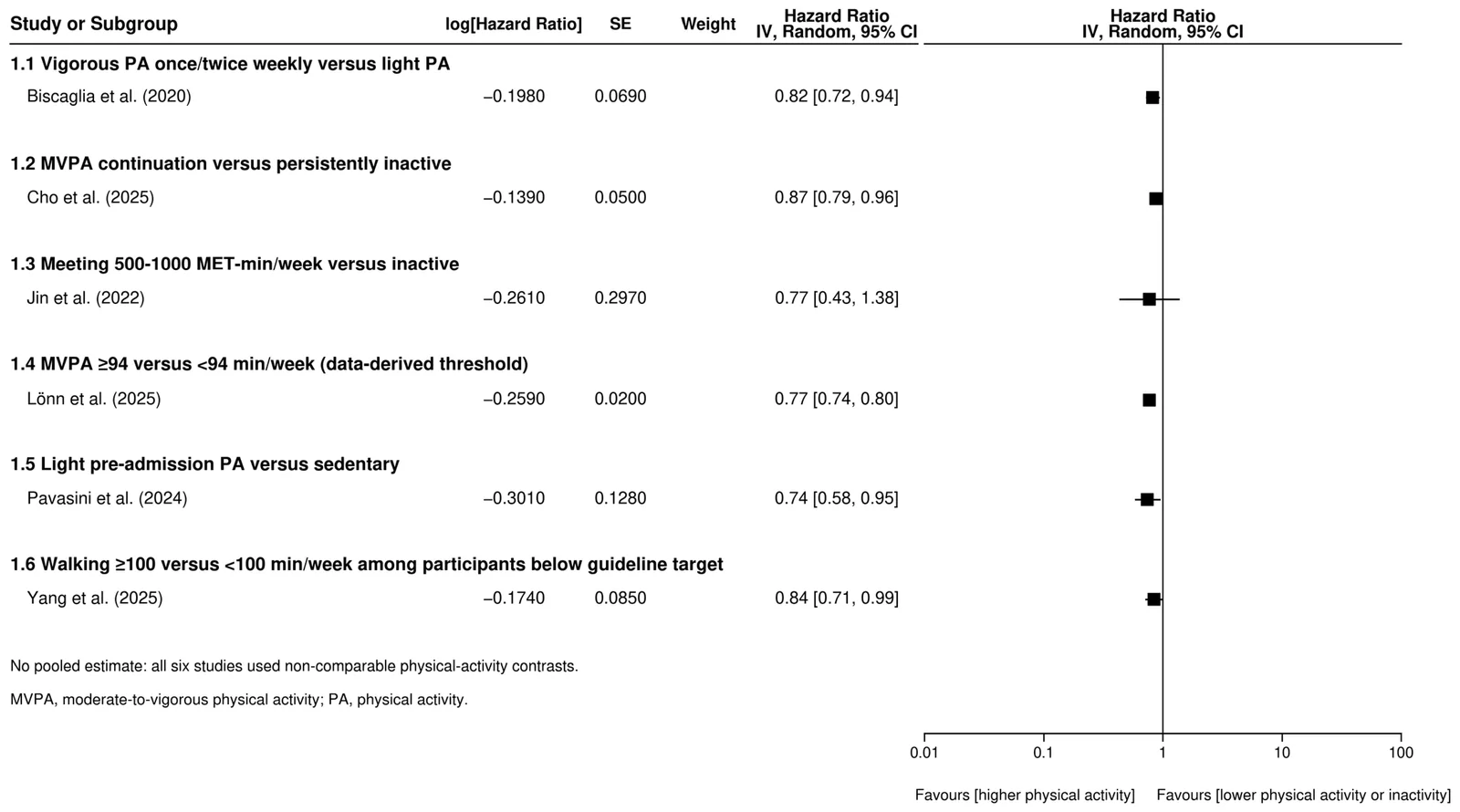
Forest plot of hazard ratios for the association of habitual physical activity with MACE in patients with coronary heart disease [50,51,55,62,75,77].

Six studies [50,51,55,62,75,77] examined the association between physical activity and MACE. All six studies used different physical-activity contrasts; therefore, no pooled estimate was calculated. Vigorous physical activity once or twice weekly, compared with light activity, was associated with a lower risk of MACE (HR = 0.82, 95% CI: 0.72 to 0.94). Maintaining moderate to vigorous physical activity before and after an acute coronary syndrome was associated with an HR of 0.87 (95% CI 0.79 to 0.96) compared with persistent inactivity. With inactive participants as the reference group, the HR for MACE at 500 to 1000 MET-min/week was 0.77 (95% CI 0.43 to 1.38), with the confidence interval including the null value. The remaining study-specific associations were HR = 0.77 (95% CI: 0.74 to 0.80) for meeting a data-derived threshold of at least 94 min/week of moderate-to-vigorous physical activity, HR = 0.74 (95% CI: 0.58 to 0.95) for light pre-admission activity versus sedentary behaviour, and HR = 0.84 (95% CI: 0.71 to 0.99) for walking at least 100 versus less than 100 min/week among participants below the overall activity target. Differences in both physical-activity contrasts and composite outcome definitions precluded quantitative pooling (Figure 7).

### 3.8. Sensitivity and Subgroup Analyses

Leave-one-out sensitivity analyses were conducted within the estimand-specific meta-analyses containing at least three studies. One study at a time was omitted, and the pooled estimate was recalculated using the generic inverse variance random effects model applied in the primary analysis.

**Figure 8:**
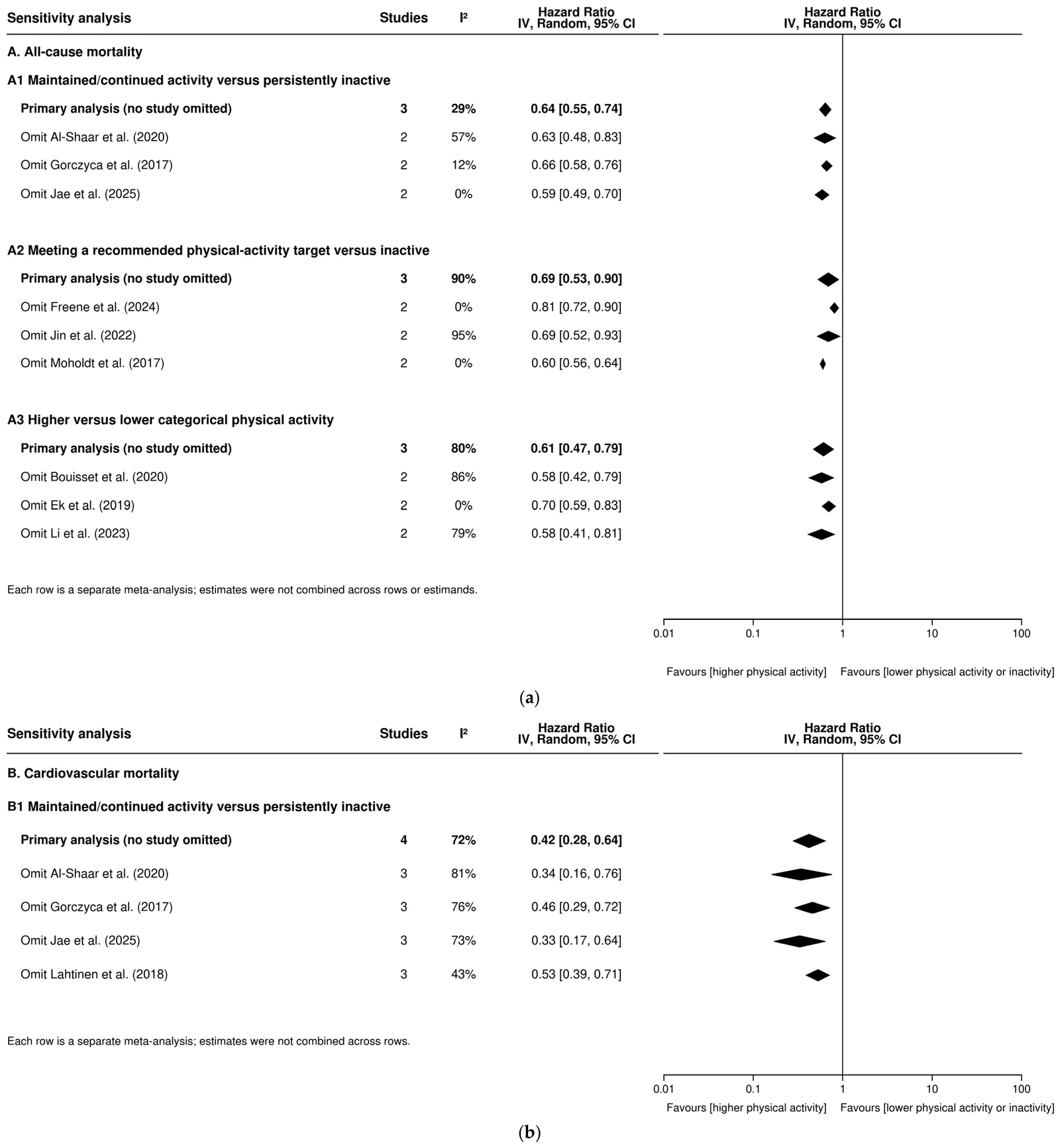
(**a**) Leave-one-out sensitivity analyses of the association between physical activity and all-cause mortality across comparable exposure estimands. (**b**) Leave-one-out sensitivity analysis of maintained or continued physical activity and cardiovascular mortality [49,51,54,57,58,59,64,65,73,74].

In the primary analysis of patients with CHD, the pooled HR for all-cause mortality was 0.64 (95% CI 0.55 to 0.74; *I*^2^ = 29%) for maintained or continued physical activity versus persistent inactivity. Omitting Al-Shaar et al. (2020) [58] yielded an HR of 0.63 (95% CI 0.48 to 0.83) and increased *I*^2^ to 57%. When Gorczyca et al. (2017) [59] was removed, the HR was 0.66 (95% CI 0.58 to 0.76), with *I*^2^ decreasing to 12%. Removal of Jae et al. (2025) [49] resulted in an HR of 0.59 (95% CI 0.49 to 0.70), with no heterogeneity detected (*I*^2^ = 0%). The association remained statistically significant in all analyses, although the degree of heterogeneity varied according to the study omitted (Figure 8a).

Compared with inactivity, meeting the recommended physical activity target yielded a pooled HR of 0.69 (95% CI 0.53 to 0.90; *I*^2^ = 90%) in the primary analysis. Omitting Freene et al. (2024) [57] resulted in an HR of 0.81 (95% CI 0.72 to 0.90), with *I*^2^ falling to 0%. Removal of Jin et al. (2022) [51] yielded an HR of 0.69 (95% CI 0.52 to 0.93), while *I*^2^ rose to 95%. After excluding Moholdt et al. (2017) [74], the summary HR was 0.60 (95% CI 0.56 to 0.64), with *I*^2^ falling to 0%. The inverse association remained statistically significant in every analysis, although its magnitude and the degree of heterogeneity varied according to the study omitted (Figure 8a).

Using the activity categories specified in each study, the pooled HR comparing greater with lesser physical activity was 0.61 (95% CI 0.47 to 0.79; *I*^2^ = 80%). The recalculated HRs after separately removing Bouisset et al. (2020) [64], Ek et al. (2019) [65], and Li et al. (2023) [54] were 0.58 (95% CI 0.42 to 0.79; *I*^2^ = 86%), 0.70 (95% CI 0.59 to 0.83; *I*^2^ = 0%), and 0.58 (95% CI 0.41 to 0.81; *I*^2^ = 79%), respectively. Every confidence interval remained below 1.00, indicating that the direction and statistical significance of the finding did not depend on any single study. Notably, *I*^2^ fell to 0% only after the removal of Ek et al. (2019) [65], identifying this study was an important contributor to variability across studies (Figure 8a).

Among patients with coronary heart disease, maintained or continued physical activity was associated with lower cardiovascular mortality than persistent inactivity in the primary analysis (HR 0.42, 95% CI 0.28 to 0.64; *I*^2^ = 72%). In the leave-one-out analysis, the summary HRs after separately excluding Al-Shaar et al. (2020) [58], Gorczyca et al. (2017) [59], Jae et al. (2025) [49] and Lahtinen et al. (2018) [73] were 0.34 (95% CI 0.16 to 0.76; *I*^2^ = 81%), 0.46 (95% CI 0.29 to 0.72; *I*^2^ = 76%), 0.33 (95% CI 0.17 to 0.64; *I*^2^ = 73%) and 0.53 (95% CI 0.39 to 0.71; *I*^2^ = 43%), respectively. Statistical significance was retained in every analysis. The marked reduction in heterogeneity following the removal of Lahtinen et al. (2018) [73] indicates that this study was the principal contributor to variability across studies (Figure 8b).

Overall, the leave-one-out analyses demonstrated that the direction and statistical significance of the associations with all-cause and cardiovascular mortality were not driven by any single study. Although the inverse associations persisted across the leave one out analyses, the pooled estimates and heterogeneity varied substantially depending on which study was omitted. Thus, the direction of the associations was stable, but their exact magnitude and consistency across studies warrant cautious interpretation.

### 3.9. Narrative Synthesis of Studies Jointly Assessing Mediterranean Diet and Physical Activity

A total of five studies [42,43,44,47,68] examined Mediterranean diet- and exercise-related lifestyle factors in relation to mortality and cardiovascular outcome on mortality or MACE among patients with coronary heart disease. Cangemi et al. reported that, among patients with ischemic heart disease, the incidence of MACE decreased progressively with increasing adherence to the Mediterranean diet, from 17.5% in the low-adherence group to 8.1% and 3.9% in the moderate- and high-adherence groups, respectively. After adjustment for age, sex, BMI, and Duke Activity Status Index scores, moderate or high adherence to the Mediterranean diet remained associated with a lower risk of MACE. In a cohort of 690 patients experiencing their first ACS episode, Kouvari et al. reported lower estimated risks of fatal and nonfatal recurrence at higher MedDietScore. This model was adjusted for physical activity and other potential confounders; however, the association was attenuated after further adjustment for left ventricular ejection fraction. Stewart et al. reported that, among patients with stable coronary heart disease, a higher Mediterranean diet score was associated with a lower risk of MACE in the fully adjusted model, which included self-reported physical activity. However, the associations with cardiovascular mortality and all-cause mortality were no longer statistically significant. Díaz-Gutiérrez et al. used the MEDLIFE index, which integrates Mediterranean diet, physical activity, rest, and social habits, to evaluate overall adherence to a Mediterranean lifestyle. They found that participants in the highest adherence group had a lower risk of adverse events than those in the lowest adherence group. Castro-Conde et al. compared intensive versus standard exercise-based cardiac rehabilitation among patients after ACS. Both groups received Mediterranean diet education and physical activity guidance. After one year, physical activity capacity and Mediterranean diet adherence improved in both groups, but no significant difference in cardiovascular event rates was observed between the two groups.

Overall, the available evidence suggests that lifestyle interventions or patterns incorporating both the Mediterranean diet and physical activity may be associated with favourable cardiovascular outcomes. However, no quantitative synthesis was undertaken, and the available evidence could not establish whether combining the Mediterranean diet with physical activity produced independent, additive, or synergistic benefits, or reduced mortality or MACE beyond either component alone.

## 4. Risk of Bias and Certainty of Evidence Assessment

Risk of bias was evaluated separately for randomized interventions, comparative non-randomized interventions, and observational exposure studies. Among the six randomized intervention reports assessed using RoB 2, one was judged to have a low overall risk of bias, three had some concerns, and two had a high risk of bias. More specifically, RoB 2 indicated a low overall risk of bias for the Mediterranean diet trial. In the exercise-based cardiac rehabilitation trials, concerns arose mainly from departures from assigned interventions, incomplete outcome data, and the selection of reported results.

Assessment with ROBINS-I V2 classified the overall risk of bias as critical in three of the four comparative nonrandomized intervention studies and as serious in the remaining study. These judgements were mainly driven by uncontrolled confounding, misalignment of intervention classification or time zero, selection into the analysis, and potential immortal-time bias.

ROBINS-E assessment classified 25 of the 30 observational exposure studies as high risk overall, with some concerns recorded for the other five. A high overall risk of bias was identified in six of nine studies examining Mediterranean diet adherence and in 19 of 21 studies evaluating physical activity, sedentary behaviour, or fitness. The main concerns involved residual confounding, self-reported exposure measurement, and selection into the study or repeated-measurement analysis. Detailed assessments are presented in Supplementary Material File S1: Figures S1–S6 [78].

In addition, the certainty of evidence was evaluated using the GRADE approach to assess the strength of evidence for the associations of the Mediterranean diet and physical activity with mortality and MACE outcomes. The detailed GRADE evidence profiles are presented in the Supplementary Material File S1: Table S1.

## 5. Assessment of Publication Bias

After stratification by outcome, study design, exposure or intervention type, and estimand, no quantitative synthesis included at least 10 independent studies. Consequently, funnel plots and Egger’s regression tests were not performed. Publication bias and small-study effects could therefore not be formally assessed.

## 6. Discussion

In the present systematic review and meta-analysis, we synthesized evidence regarding the effects of the Mediterranean diet, physical activity, and combined lifestyle patterns on all-cause mortality, cardiovascular mortality, and MACE among patients with CHD. Our findings indicate that, among individuals with established CHD, greater adherence to the Mediterranean diet was associated with lower all-cause mortality, although its association with cardiovascular mortality remained uncertain. Evidence from both interventions trials and observational studies generally favored a beneficial association between Mediterranean diet adherence and reduced risk of MACE. Similarly, maintaining or achieving higher levels of physical activity was associated with lower all-cause and cardiovascular mortality, with findings for MACE showing a generally favorable direction. However, evidence regarding combined diet-and-physical-activity lifestyle patterns remained limited. Collectively, these findings suggest that optimizing dietary patterns and regular physical activity may represent important lifestyle-based strategies for improving long-term outcomes in the secondary prevention of coronary heart disease.

To date, several comprehensive reviews and meta-analyses have investigated the associations of the Mediterranean diet and lifestyle factors with cardiovascular outcomes [19,79,80]. However, most prior studies have focused on cardiovascular disease as a broad clinical category [81], and evidence specifically focusing on patients with established CHD remains relatively limited. Furthermore, few studies have comprehensively evaluated the combined contributions of the Mediterranean diet and physical activity to mortality and MACE risk in this high-risk population. Our findings extend the existing evidence by demonstrating that, lifestyle modification may remain clinically relevant even after the development of CHD. In secondary prevention, greater adherence to a Mediterranean dietary pattern may be associated with lower risks of all-cause mortality and MACE.

The Mediterranean diet has gained broad recognition as an eating pattern associated with numerous health benefits [82]. Its potential role in cardiovascular prevention has also been recognized by international public health organizations, including the World Health Organization [83]. Consistent with our findings, a previous meta-analysis reported that greater adherence to the Mediterranean diet was associated with improved survival among individuals with a history of cardiovascular disease [80]. Sebastian et al. likewise identified significant inverse associations of this dietary pattern with MACE, myocardial infarction, stroke, and cardiovascular mortality in individuals at elevated cardiovascular risk [84]. However, the estimate for all-cause mortality did not reach statistical significance.

The beneficial effects of the Mediterranean diet in patients with cardiovascular disease may be attributable to multiple components of this dietary pattern, including higher intakes of fruits, vegetables, nuts, and fish [85]. Greater consumption of these foods has been linked to lower inflammatory activity and oxidative burden, both of which are implicated in cardiovascular disease pathogenesis [86,87,88]. A recent randomized controlled trial also found that closer adherence to a Mediterranean dietary pattern was linked to enhanced endothelial function and beneficial changes in high density lipoprotein cholesterol, fasting glucose, and high sensitivity C reactive protein [89]. These findings suggest that the Mediterranean dietary pattern may exert protective and health-promoting effects partly through improvements in metabolic profiles. In addition, a meta-analysis of prospective studies showed that higher intake of individual components of the Mediterranean diet, such as whole grains, vegetables, fruits, nuts, and fish, was associated with a lower risk of all-cause mortality [90]. Therefore, considering the Mediterranean diet as an overall dietary pattern rather than as a single-nutrient intervention may better capture the synergistic effects among different food components. Such synergistic effects may not only improve metabolic indicators but also contribute to a lower risk of mortality.

Our meta-analysis also showed that maintaining or achieving higher levels of physical activity was associated with lower all-cause, cardiovascular mortality and MACE. These findings are consistent with previous evidence indicating that physical activity may improve mortality-related outcomes in patients with coronary heart disease [79]. Regular physical activity and exercise-based cardiac rehabilitation may improve cardiorespiratory fitness [91], enhance autonomic regulation [92], reduce resting heart rate and blood pressure, and lower cardiovascular burden through anti-inflammatory effects [93], improved endothelial function [94], and enhanced skeletal muscle metabolic capacity [95]. Through these interrelated mechanisms, physical activity plays an important role in improving cardiac health and slowing disease progression, thereby reducing the occurrence of adverse cardiovascular events. However, our study also identified several inconsistencies. Although individuals who remained a higher level of physical activity had a lower risk of mortality, the definitions of physical activity varied substantially across studies, including baseline physical activity level, changes in physical activity over time, and cardiac rehabilitation programs involving different types or intensities of physical activity. This interpretation is also supported by the systematic review by Arita et al., which examined the Mediterranean diet, physical activity, and combined lifestyle interventions and highlighted substantial variability in intervention components, duration, adherence, and outcome assessment across studies. These differences underscore the importance of standardized reporting to enhance the robustness and comparability of future evidence [96].

Our findings also provide insights into the current evidence regarding lifestyle approaches that jointly incorporating the Mediterranean diet and physical activity in the secondary prevention of CHD. Evidence evaluating combined lifestyle approaches jointly incorporating the Mediterranean diet and physical activity was sparse and of very low certainty. Therefore, these heterogeneous narrative findings should be interpreted as hypothesis-generating and do not establish an independent, additive, or synergistic benefit of combining the Mediterranean diet with physical activity. Given the increasing global burden of CHD and its persistent adverse long-term prognosis [6,7], integrated lifestyle strategies represent promising and clinically relevant non-pharmacological approaches for disease management. However, future factorial or component-specific trials are needed to distinguish the individual and interactive effects of these lifestyle components. Such evidence may improve adherence to healthy behaviors and ultimately enhance outcomes among patients with CHD.

The interpretation of our findings varied according to study design and the certainty of available evidence. One randomized trial provided moderate-certainty evidence that a Mediterranean diet intervention reduced MACE, whereas evidence from randomized trials regarding cardiovascular mortality was of low certainty and evidence for structured physical activity or cardiac rehabilitation was of very low certainty. In contrast, observational studies suggested that greater adherence to the Mediterranean diet was associated with lower all-cause mortality and that higher levels of habitual physical activity was associated with reduced all-cause and cardiovascular mortality. Associations with MACE were generally favorable but heterogeneous, and evidence for combined lifestyle patterns remained sparse. All observational evidence was rated as very low certainty.

These observational associations should be interpreted cautiously because of residual confounding and reverse causation. Patients with less severe disease, better functional capacity, and lower frailty may be more active and independently have a better prognosis, whereas worsening health may itself reduce physical activity. Healthy-user bias may similarly influence Mediterranean diet adherence. A contemporary meta-analysis of randomized trials reported an RR of 0.96 for all-cause mortality and 0.74 for cardiovascular mortality at the longest follow-up [79]. This discrepancy may partly reflect residual confounding and reverse causation, although differences in estimands and follow-up also preclude direct numerical comparison.

Although pooling was restricted to clinically aligned exposure contrasts, heterogeneity remained extreme for meeting a recommended physical-activity target in relation to all-cause mortality (*I*^2^ = 90%) and cardiovascular mortality (*I*^2^ = 91%) and was also substantial for other contrasts. These differences may reflect variation in activity definitions, assessment periods, disease severity, functional capacity, adjustment strategies, and patient characteristics. Leave-one-out analyses preserved the direction of the associations but did not consistently resolve the heterogeneity. Consequently, each pooled estimate should be interpreted as an average association within a specific exposure contrast and should not be generalized to a particular physical activity intervention, physical-activity definition, or subgroup of patients with coronary heart disease.

This study has several strengths. First, we focused on the secondary prevention setting among patients with established coronary heart disease rather than on primary prevention in the general population, thereby enhancing the clinical relevance of the study population. Second, it incorporates recently published evidence identified through 15 June 2026. Third, it separately examines Mediterranean diet interventions, Mediterranean diet adherence, structured physical activity or exercise-based cardiac rehabilitation, with findings interpreted according to study design and clinically distinct exposure contrasts. Finally, it evaluates interventions and lifestyle indices jointly incorporating diet and physical activity. Therefore, including and distinguishing these interventions better reflects real-world clinical practice and may help clarify whether the Mediterranean diet and physical activity have complementary or synergistic effects.

Nevertheless, several limitations should be acknowledged. First, a substantial proportion of the included studies were observational cohort studies. Despite multivariable adjustment, residual confounding by socioeconomic status, medication adherence, healthcare access, and other healthy-user characteristics, together with reverse causation, cannot be excluded. In particular, lifestyle interventions involving diet and physical activity showed marked heterogeneity in design and implementation, which posed a major challenge for evidence synthesis. Second, Mediterranean diet and physical activity exposures were largely based on self-reported measures, which are susceptible to recall bias and measurement error. Moreover, assessment tools for Mediterranean diet adherence and physical activity levels varied across studies. Third, the number of studies included for some outcomes was limited, reducing the statistical power of subgroup and sensitivity analyses; therefore, these findings should be interpreted as exploratory. Finally, potential interactions between the Mediterranean diet and physical activity may exist, but few available studies directly reported the independent and interactive effects of combined Mediterranean diet and physical activity interventions on mortality or MACE among patients with coronary heart disease. These limitations highlight the need for future studies to improve reporting of such interventions, harmonize Mediterranean diet and physical activity protocols, apply standardized assessment tools, and conduct large-scale, high-quality randomized controlled trials to better clarify the effects of combined Mediterranean diet and exercise-based lifestyle interventions on clinically relevant outcomes in patients with coronary heart disease.

## 7. Conclusions

In conclusion, greater adherence to the Mediterranean diet was associated with lower all-cause mortality among patients with CHD, although its association with cardiovascular mortality remained uncertain. Mediterranean dietary patterns were generally associated with a lower risk of MACE. Similarly, maintaining or achieving higher physical activity was associated with lower all-cause and cardiovascular mortality, while findings for MACE were generally favorable. However, evidence from combined Mediterranean diet and physical activity strategies remains limited and was derived predominantly from observational studies. Future well-designed, adequately powered, and long-term randomized studies are needed to determine the causal effects of these lifestyle interventions and to determine whether combined Mediterranean diet and physical activity provide additional benefits beyond either approach alone.

## Figures and Tables

**Table 1:** Characteristics of included studies evaluating Mediterranean diet interventions or adherence.

Study	Country	Design	Participants Demographics	No. of Participants	Age	Duration (Years)	Intervention(s) Description	Control Description	Reported Outcome(s)
Delgado-Lista et al., (2022) [21]	Spain	RCT	Patients with coronary heart disease	1002	59.5 ± 8.7 years	7.0	Mediterranean diet	Low-fat diet	MACE; Cardiac mortality
Cangemi et al., (2023) [43]	Italy	Prospective observational study	Patients with ischemic heart disease	503	66.8 ± 11.2 years	1.83	High Mediterranean diet adherence	Low Mediterranean diet adherence	MACE; Cardiac mortality; Non-fatal recurrent cardiovascular events
Shikany et al., (2018) [39]	United States	Prospective cohort study	Patients with coronary heart disease	3562	68.6 ± 8.9 years	7.1	High Mediterranean diet score	Low Mediterranean diet score	All-cause mortality; Cardiac mortality; Non-fatal recurrent cardiovascular events
Díaz-Gutiérrez et al., (2026) [42]	Spain	Prospective pilot cohort study	Patients with ischemic heart disease and/or atrial fibrillation	115	66.9 ± 11.6 years	1.38	High Mediterranean lifestyle adherence	Low Mediterranean lifestyle adherence	All-cause mortality; MACE; Non-fatal recurrent cardiovascular events
Lau et al., (2015) [45]	Hong Kong, China	Prospective observational study	Patients with stable coronary artery disease	274	68 ± 10 years	6.4	High Mediterranean diet score	Low Mediterranean diet score	MACE; All-cause mortality; Cardiac mortality; Stroke
Kouvari et al., (2017) [44]	Greece	Prospective observational study	Patients with first-diagnosed acute coronary syndrome	690	62 ± 12 years	10.0	High Mediterranean diet score	Low Mediterranean diet score	Cardiac mortality; Non-fatal recurrent cardiovascular events; ACS recurrence
Magnoni et al., (2020) [46]	Italy, Scotland, and other European regions	Prospective observational study	Patients with first STEMI	533	61.1 ± 11.7 years	0.5	High Mediterranean diet score	Low Mediterranean diet score	MACE; All-cause mortality; Non-fatal recurrent cardiovascular events
Liang et al., (2022) [40]	United States	NHANES-based cohort study	Patients with coronary heart disease or stroke	2052	65.1 years	5.6	High aMED score	Low aMED score	All-cause mortality; Cardiac mortality; Cancer mortality
Pan et al., (2025) [41]	United States	NHANES-based cohort study	Patients with coronary heart disease	3088	Mostly ≥60 years	5.75	High healthy dietary index/Mediterranean diet score	Low healthy dietary index/Mediterranean diet score	All-cause mortality; Cardiac mortality; Cause-specific mortality
Stewart et al., (2016) [47]	39 countries	Prospective observational study	Patients with stable coronary heart disease	15,482	64.2 ± 9.5 years	3.7	High Mediterranean diet score	Low Mediterranean diet score	MACE; All-cause mortality; Cardiac mortality; Non-fatal recurrent cardiovascular events

Abbreviations: ACS, acute coronary syndrome; aMED, alternative Mediterranean diet score; MACE, major adverse cardiovascular events; NHANES, National Health and Nutrition Examination Survey; RCT, randomized controlled trial; STEMI, ST-segment elevation myocardial infarction.

**Table 2:** Characteristics of included studies evaluating structured physical activity, exercise-based cardiac rehabilitation, physical activity, sedentary behaviour, or cardiorespiratory fitness.

Study	Country	Design	Participants Demographics	No. of Participants	Age	Duration (Years)	Intervention(s) Description	Control Description	Reported Outcome(s)
Seron et al., (2024) [67]	Chile	RCT	Patients with coronary artery disease	191	58.74 ± 9.80 years	1.0	Exercise-based hybrid cardiac rehabilitation	Exercise-based standard cardiac rehabilitation	MACE; All-cause mortality
Castro-Conde et al., (2021) [68]	Spain	RCT	Patients after acute coronary syndrome	497	57.8 ± 10.0 years	1.0	Exercise-based intensive cardiac rehabilitation	Exercise-based standard cardiac rehabilitation	MACE; All-cause mortality; Cardiac mortality
Pavasini et al., (2024) [75]	Italy and Spain	Observational exposure analysis nested within a randomized trial	Older patients with myocardial infarction and multivessel disease	1445	≥75 years	1.0	Pre-admission physical activity	Sedentary lifestyle/no physical activity	MACE; All-cause mortality; Cardiac mortality; Non-fatal recurrent cardiovascular events
Tüner et al., (2025) [69]	Turkey	RCT	Patients with acute coronary syndrome	340	63.8 ± 9.3 years	1.0	Exercise-based cardiac rehabilitation	Standard recommendations	Hospital readmission; Revascularization; All-cause mortality
He et al., (2020) [52]	China	RCT	Patients with MINOCA	524	60.8 ± 12.8 years	3.0	Exercise-based cardiac rehabilitation	No exercise-based cardiac rehabilitation	MACE; All-cause mortality
Chaves et al., (2019) [70]	Brazil	Wait-list control crossover RCT	Patients with coronary artery disease	115	59.5 ± 9.4 years	1.0	Exercise-based comprehensive cardiac rehabilitation/exercise-only cardiac rehabilitation	Wait-list control	All-cause mortality; Non-fatal recurrent cardiovascular events; Hospitalization
Wu et al., (2024) [53]	China	Non-randomized comparative study	Patients with stable angina and severe coronary artery stenosis	100	64–65 years	1.0	OMT plus exercise-based cardiac rehabilitation	OMT plus PCI	MACE; Cardiac mortality; Non-fatal recurrent cardiovascular events; Angina recurrence
Taylor et al., (2016) [61]	United Kingdom	Retrospective cohort study	Patients with coronary heart disease	670	22–82 years	14.0	Higher submaximal cardiorespiratory fitness after exercise-based rehabilitation	Lower submaximal cardiorespiratory fitness after exercise-based rehabilitation	All-cause mortality
Hiruma et al., (2024) [71]	Japan	Retrospective cohort study	Patients with acute myocardial infarction	610	66–68 years	6.1 ± 4.0	Exercise-based comprehensive cardiac rehabilitation	No cardiac rehabilitation	MACE; Cardiac mortality; Non-fatal recurrent cardiovascular events; Heart failure hospitalization
Lin et al., (2016) [72]	Taiwan, China	Nationwide retrospective cohort study	Patients with acute myocardial infarction receiving Phase I cardiac rehabilitation	6713	65.52 ± 12.71 years	1.0	Female patients receiving exercise-based Phase I cardiac rehabilitation	Male patients receiving exercise-based Phase I cardiac rehabilitation	All-cause mortality; In-hospital mortality; 30-day mortality
Guy et al., (2019) [63]	France	Retrospective multicenter observational study	Male patients with coronary artery disease after PCI	108	57.3 ± 9.1 years	4.8	Intensive leisure-time sport/competitive sport after PCI	Moderate leisure-time sport	MACE; Non-fatal recurrent cardiovascular events; Cardiac mortality
Ek et al., (2019) [65]	Sweden	Nationwide registry-based cohort study	Patients after myocardial infarction	30,614	18–74 years	3.58	Medium/high physical activity after MI	Low physical activity after MI	All-cause mortality; Hospital readmission
Li et al., (2023) [54]	China	Nationwide population-based cohort study	Myocardial infarction survivors	20,653	35–75 years	3.7	Sufficient physical activity	Insufficient physical activity	All-cause mortality
Buckley et al., (2022) [60]	Predominantly United States/TriNetX global network	Retrospective cohort study	Patients with chronic coronary syndrome	18,383	≥18 years	1.5	Exercise-based cardiac rehabilitation	PCI	All-cause mortality; Hospital readmission; Non-fatal recurrent cardiovascular events; Heart failure
Lee et al., (2016) [48]	South Korea	Prospective registry analysis	Patients with left main coronary artery stenosis	3040	Not clearly reported	7.0	Exercise-based cardiac rehabilitation	No cardiac rehabilitation	All-cause mortality; Cardiac mortality; Non-fatal recurrent cardiovascular events; Stroke
Lönn et al., (2023) [66]	Sweden	Nationwide registry-based cohort study	Myocardial infarction survivors	47,153	18–79 years	6.0	Remaining physically active/increased physical activity after MI	Remaining physically inactive	Non-fatal recurrent cardiovascular events; Recurrent MI; Ischemic stroke; Vascular dementia
Yang et al., (2025) [62]	United Kingdom	UK Biobank prospective cohort study	Patients with coronary artery disease	19,074	40–69 years	13.5	Higher total physical activity/MVPA/walking	Lower physical activity	All-cause mortality; MACE; Cardiac mortality; Non-fatal recurrent cardiovascular events
Lönn et al., (2025) [55]	Australia	Prospective cohort study	Patients with coronary heart disease	40,156	70 years	8.3	Meeting MVPA threshold/lower sedentary behaviour	Not meeting MVPA threshold/higher sedentary behaviour	MACE; Non-fatal recurrent cardiovascular events; Cardiac mortality
Lönn et al., (2025) [56]	Australia	Prospective cohort study	Patients with coronary heart disease	9430	70 ± 10 years	4.9	Remaining high or increased MVPA/low or decreased sedentary behaviour	Remaining low MVPA/high sedentary behaviour	MACE; Non-fatal recurrent cardiovascular events; Cardiac mortality
Jae et al., (2025) [49]	South Korea	Nationwide cohort study	Patients after acute coronary syndrome	30,840	60 years	5.8	MVPA initiation/MVPA continuation after ACS	Persistently inactive	All-cause mortality; Cardiac mortality
Cho et al., (2025) [50]	South Korea	Nationwide longitudinal cohort study	Patients with acute coronary syndrome	30,840	60 ± 11 years	6.7	Increased physical activity after ACS/MVPA initiation or continuation	Persistently inactive	MACE; Cardiac mortality; Non-fatal coronary events; Non-fatal stroke
Freene et al., (2024) [57]	Australia	Prospective cohort study	Patients with coronary heart disease	40,156	70.3 ± 10.3 years	11.1	Higher physical activity/MVPA/walking; lower sedentary behavior	No physical activity/higher sedentary behavior	All-cause mortality; Cardiac mortality
Al-Shaar et al., (2020) [58]	United States	Prospective cohort study	Male myocardial infarction survivors	1651	40–75 years	14.0	Maintained high physical activity/increased physical activity after MI/walking	Maintained low physical activity	All-cause mortality; Cardiac mortality
Gorczyca et al., (2017) [59]	United States	Prospective cohort study	Postmenopausal women after myocardial infarction	856; 533 for sitting-time analysis	50–79 years	7.2	Increased or maintained high physical activity after MI/lower sitting time	Maintained low physical activity/higher sitting time	All-cause mortality; CHD mortality; CVD mortality
Biscaglia et al., (2020) [77]	45 countries	International prospective registry study	Patients with stable coronary artery disease	32,370	Not clearly reported	5.0	Light or vigorous physical activity	Sedentary/low physical activity	MACE; All-cause mortality; Cardiac mortality; Stroke
Lahtinen et al., (2018) [73]	Finland	Prospective cohort study	Patients with stable coronary artery disease	1746	Not clearly reported	4.5	Maintained at least irregular physical activity/increased physical activity	Persistently inactive/became inactive	Cardiac mortality
Jin et al., (2022) [51]	South Korea	Single-center registry cohort study	Patients with concomitant atrial fibrillation and coronary artery disease	551	67.1 ± 9.8 years	4.0	Recommended or higher physical activity level	Physical inactivity	MACCE; All-cause mortality
Moholdt et al., (2017) [74]	Norway	Prospective population-based cohort study	Patients with coronary heart disease	6493	Not clearly reported	12.5	Recommended or higher physical activity level	Physical inactivity	All-cause mortality; Cardiac mortality
Stewart et al., (2017) [76]	39 countries	Prospective observational study	Patients with stable coronary heart disease	15,486	64.2 ± 9.5 years	3.7	Higher habitual physical activity	Lower physical activity/sedentary lifestyle	All-cause mortality; Cardiac mortality; Myocardial infarction; Stroke
Bouisset et al., (2020) [64]	France	Prospective cohort study	Male patients with stable coronary heart disease	822	45–74 years	14.6	Moderate/high physical activity level	Low physical activity level	All-cause mortality

Abbreviations: ACS, acute coronary syndrome; CHD, coronary heart disease; CVD, cardiovascular disease. MACCE, major adverse cardiac and cerebrovascular events; MACE, major adverse cardiovascular events; MI, myocardial infarction; MINOCA, myocardial infarction with non-obstructive coronary arteries; MVPA, moderate-to-vigorous physical activity; OMT, optimal medical therapy; PCI, percutaneous coronary intervention; RCT, randomized controlled trial.

## Data Availability

No new data were created or analyzed in this study. Data sharing is not applicable to this article.

## Supplementary Materials

The following supporting information can be downloaded at: https://www.mdpi.com/article/10.3390/nu18162589/s1, Supplementary Material File S1: Supplementary Figures S1–S6: Supplementary Figure S1. Traffic-light plot of risk-of-bias judgements for randomized intervention studies assessed using RoB 2; Supplementary Figure S2. Summary plot of risk-of-bias judgements for randomized intervention studies assessed using RoB 2; Supplementary Figure S3. Traffic-light plot of risk-of-bias judgements for comparative non-randomized intervention studies of structured exercise or exercise-based cardiac rehabilitation assessed using ROBINS-I V2; Supplementary Figure S4. Summary plot of risk-of-bias judgements for comparative non-randomized intervention studies assessed using ROBINS-I V2; Supplementary Figure S5. Traffic-light plot of risk-of-bias judgements for observational exposure studies assessed using ROBINS-E; Supplementary Figure S6. Summary plot of risk-of-bias judgements for observational exposure studies assessed using ROBINS-E; Supplementary Tables S1–S5: Supplementary Table S1. GRADE evidence profile for Mediterranean diet interventions and adherence, structured exercise or exercise-based cardiac rehabilitation, habitual physical activity, and joint lifestyle exposures in adults with established coronary heart disease; Supplementary Table S2. Study-specific definitions and components of major adverse cardiovascular events and other composite cardiovascular outcomes; Supplementary Table S3. Report-to-parent-cohort mapping and management of overlapping populations; Supplementary Table S4. Study-level assessment methods and contribution to outcome-specific quantitative or narrative synthesis; Supplementary Table S5. Timing of Mediterranean diet or lifestyle assessment relative to coronary heart disease and consideration of secondary-prevention treatment; Supplementary Material File S2: Search strategy.

## Abbreviations

ACS, acute coronary syndrome; aMED, alternative Mediterranean diet score; BMI, body mass index; CAD, coronary artery disease; CENTRAL, Cochrane Central Register of Controlled Trials; CHD, coronary heart disease; CI, confidence interval; CR, cardiac rehabilitation; CVD, cardiovascular disease; GRADE, Grading of Recommendations, Assessment, Development and Evaluation; HR, hazard ratio; MACE, major adverse cardiovascular events; MACCE, major adverse cardiac and cerebrovascular events; MD, Mediterranean diet; MEDLIFE, Mediterranean lifestyle index; MI, myocardial infarction; MINOCA, myocardial infarction with non-obstructive coronary arteries; MVPA, moderate-to-vigorous physical activity; NHANES, National Health and Nutrition Examination Survey; OMT, optimal medical therapy; OR, odds ratio; PA, physical activity; PCI, percutaneous coronary intervention; PICOS, population, intervention, comparison, outcomes, and study design; PRISMA, Preferred Reporting Items for Systematic Reviews and Meta-Analyses; PROSPERO, International Prospective Register of Systematic Reviews; RCT, randomized controlled trial; RevMan, Review Manager; RoB 2, revised Cochrane Risk of Bias tool for randomized trials; ROBINS-I V2, Risk Of Bias In Non-randomized Studies of Interventions, version 2; RR, risk ratio; SB, sedentary behavior; SE, standard error; SMD, standardized mean difference; STEMI, ST-segment elevation myocardial infarction; WHO, World Health Organization.
