# Supplementary material for: Association of the Mediterranean Diet, Physical Activity and Related Lifestyle Factors on the Mortality and Major Adverse Cardiovascular Events in Patients with Coronary Heart Disease: A Systematic Review and Meta-Analysis
Source: Nutrients. 2026 Aug 7;18(16):2589. doi: 10.3390/nu18162589 (PMC13516061; doi:10.3390/nu18162589)
Supplement: Supplementary file 1 [file nutrients-18-02589-s001.zip › Supplementary Material File S1.pdf]

Supplementary Material File S1

Association of the Mediterranean Diet, Physical Activity and Related Lifestyle Factors on the Mortality and Major Adverse Cardiovascular Events in Patients with Coronary Heart Disease: A Systematic Review and Meta-Analysis

CATALOGUE

Supplementary Figure S1. 2

Supplementary Figure S2. 3

Supplementary Figure S3. 4

Supplementary Figure S4. 5

Supplementary Figure S5. 6

Supplementary Figure S6. 8

Supplementary Table S1. 10

Supplementary Table S2. 20

Supplementary Table S3. 23

Supplementary Table S4. 28

Supplementary Table S5. 40

Supplementary Figure S1.

Traffic-light plot of risk-of-bias judgements for randomized intervention studies assessed using RoB 2.

(a)

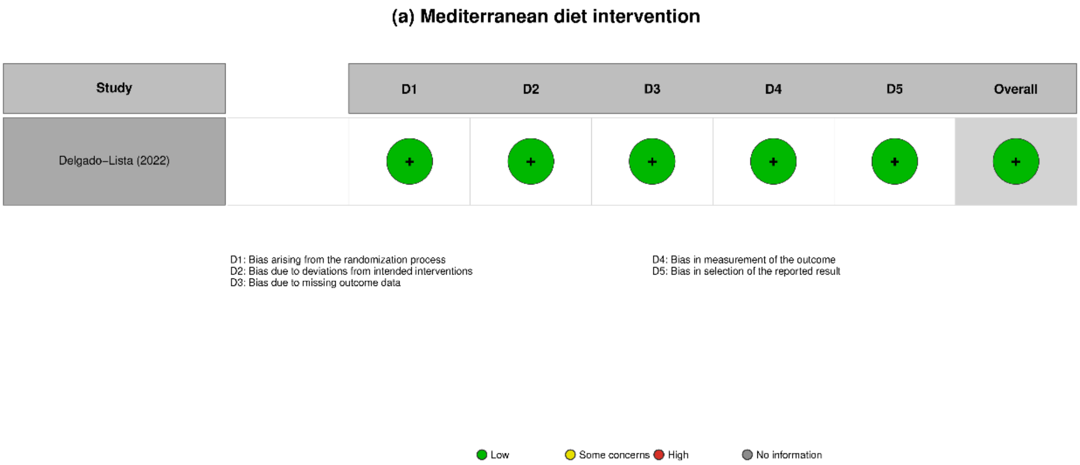

(b)

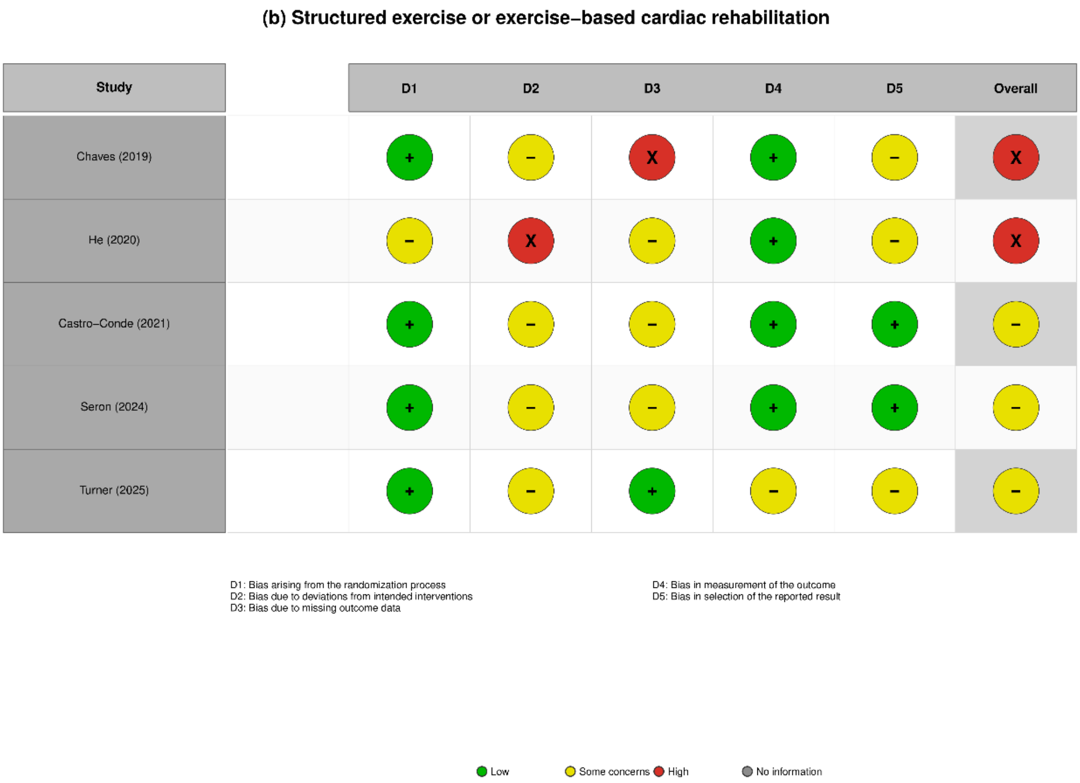

(a) Mediterranean diet intervention and (b) structured exercise or exercise-based cardiac rehabilitation.

Supplementary Figure S2.

Summary plot of risk-of-bias judgements for randomized intervention studies assessed using RoB 2.

(a)

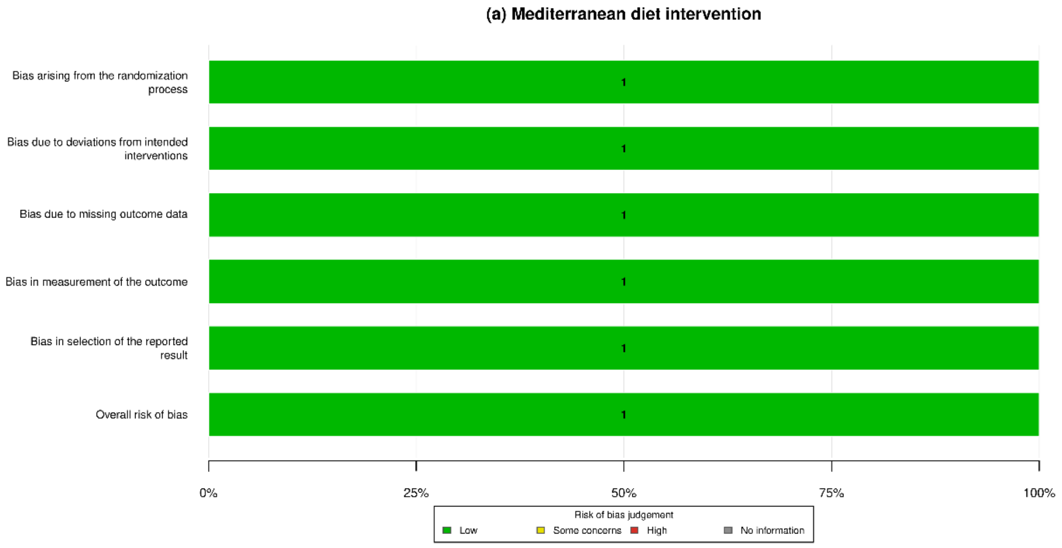

(b)

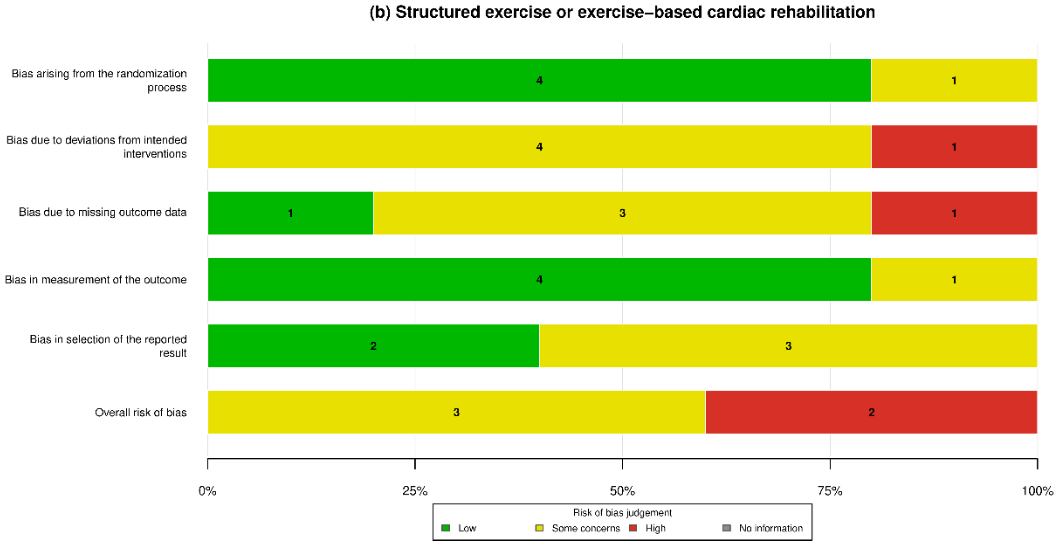

(a) Mediterranean diet intervention and (b) structured exercise or exercise-based cardiac rehabilitation.

Supplementary Figure S3.

Traffic-light plot of risk-of-bias judgements for comparative non-randomized intervention studies of structured exercise or exercise-based cardiac rehabilitation assessed using ROBINS-I V2.

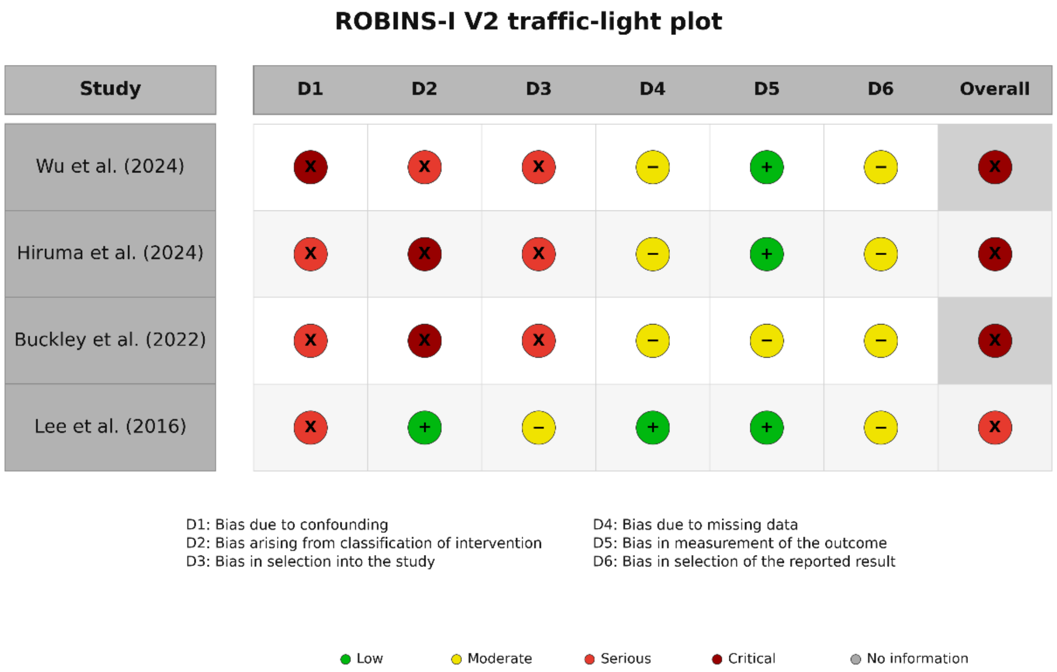

Supplementary Figure S4.

Summary plot of risk-of-bias judgements for comparative non-randomized intervention studies assessed using ROBINS-I V2.

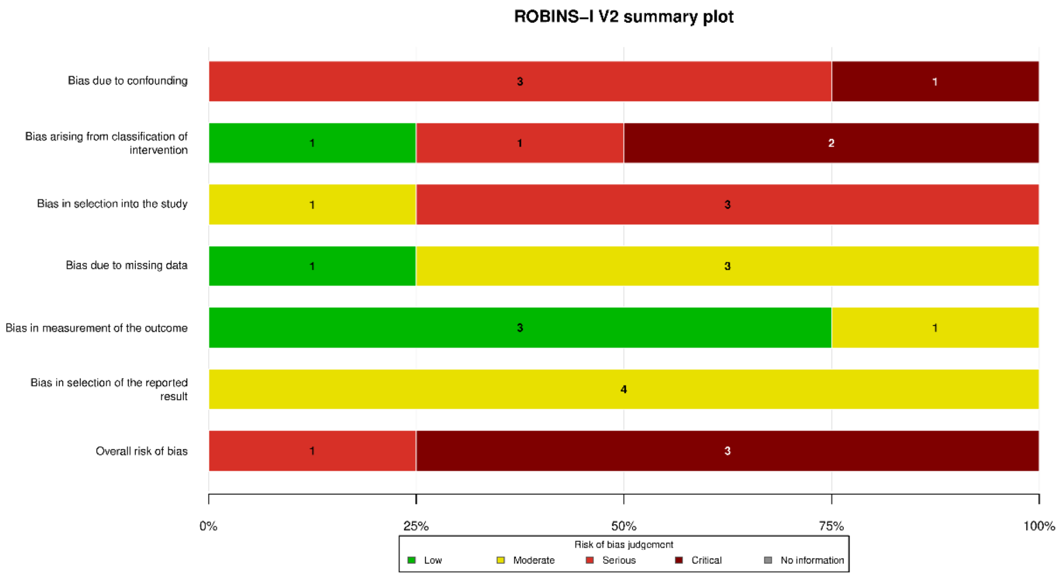

Supplementary Figure S5.

Traffic-light plot of risk-of-bias judgements for observational exposure studies assessed using ROBINS-E.

(a)

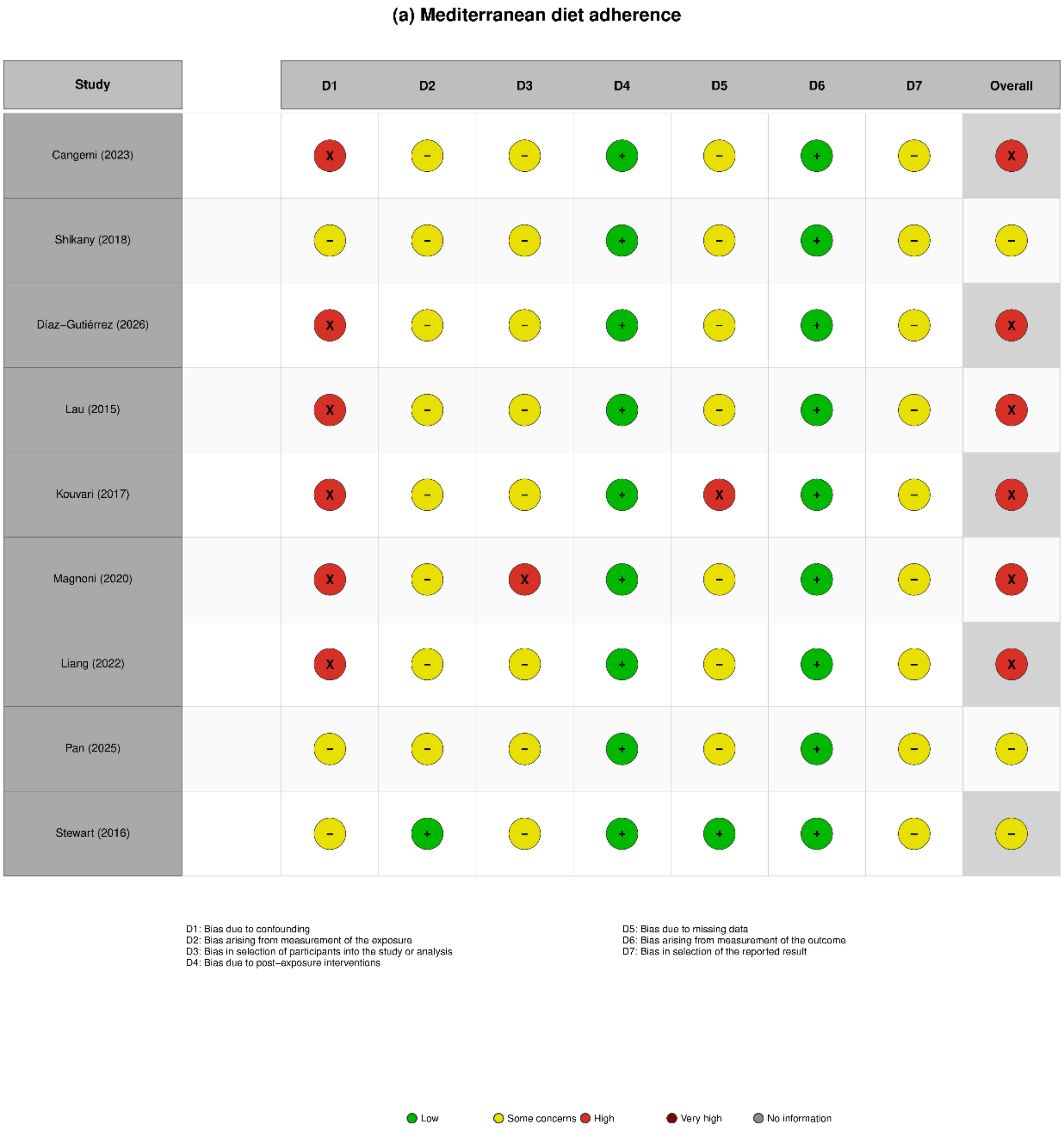

(b)

(b) Physical activity, sedentary behaviour, or fitness

Study	D1	D2	D3	D4	D5	D6	D7	Overall
Pavasini (2024)	X	–	–	+	+	+	–	X
Taylor (2016)	–	–	X	+	–	+	–	X
Lin (2016)	–	–	+	+	–	+	–	–
Guy (2019)	X	X	–	+	–	–	–	X
Ek (2019)	X	–	–	+	–	+	–	X
Li (2023)	X	–	–	+	–	+	–	X
Lönn (2023)	X	–	X	–	–	+	–	X
Yang (2025)	X	–	–	+	–	+	–	X
Lönn (2025a)	X	–	–	+	–	+	–	X
Lönn (2025b)	X	–	X	–	–	+	–	X
Jae (2025)	X	–	–	+	–	+	–	X
Cho (2025)	X	–	–	+	–	+	–	X
Freene (2024)	X	–	–	+	–	+	–	X
Al–Shaar (2020)	X	–	–	+	–	+	–	X
Gorczyca (2017)	X	X	–	+	–	+	–	X
Biscaglia (2020)	X	–	–	+	–	+	–	X
Lahtinen (2018)	–	–	–	+	–	+	–	–
Jin (2022)	X	–	–	+	–	–	–	X
Moholdt (2017)	X	–	–	+	–	+	–	X
Stewart (2017)	X	–	–	+	–	+	–	X
Bouisset (2020)	X	–	–	+	–	+	–	X

D1: Bias due to confounding
D2: Bias arising from measurement of the exposure
D3: Bias in selection of participants into the study or analysis
D4: Bias due to post–exposure interventions
D5: Bias due to missing data
D6: Bias arising from measurement of the outcome
D7: Bias in selection of the reported result

Low   Some concerns   High   Very high   No information

(a) Mediterranean diet adherence and (b) habitual physical activity, sedentary behaviour, or cardiorespiratory fitness.

Supplementary Figure S6.

Summary plot of risk-of-bias judgements for observational exposure studies assessed using ROBINS-E.

(a)

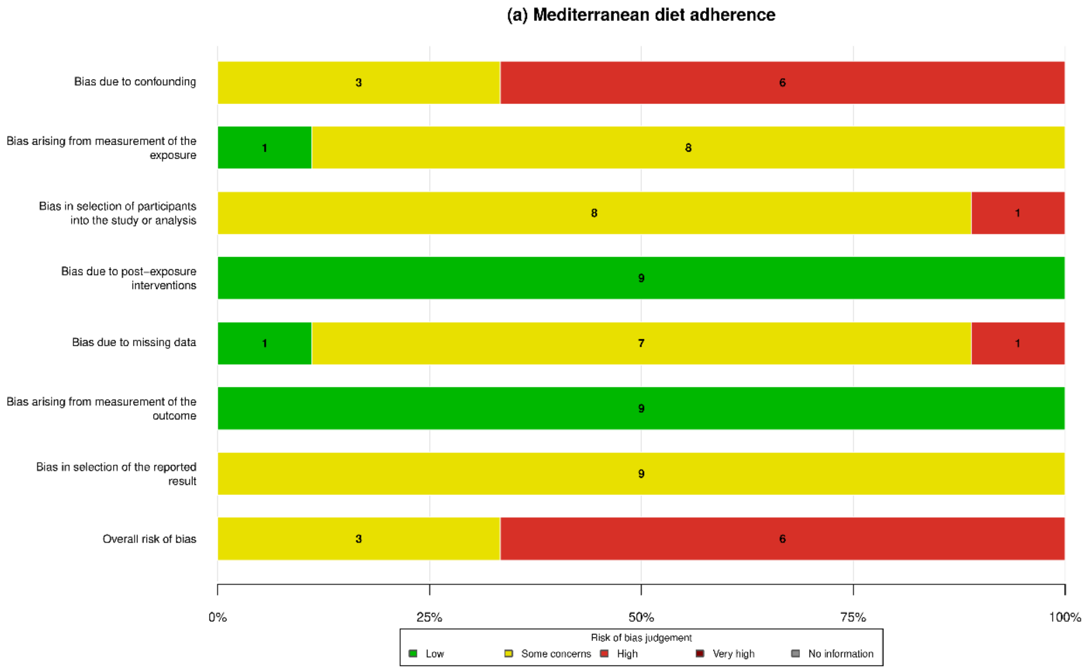

(b)

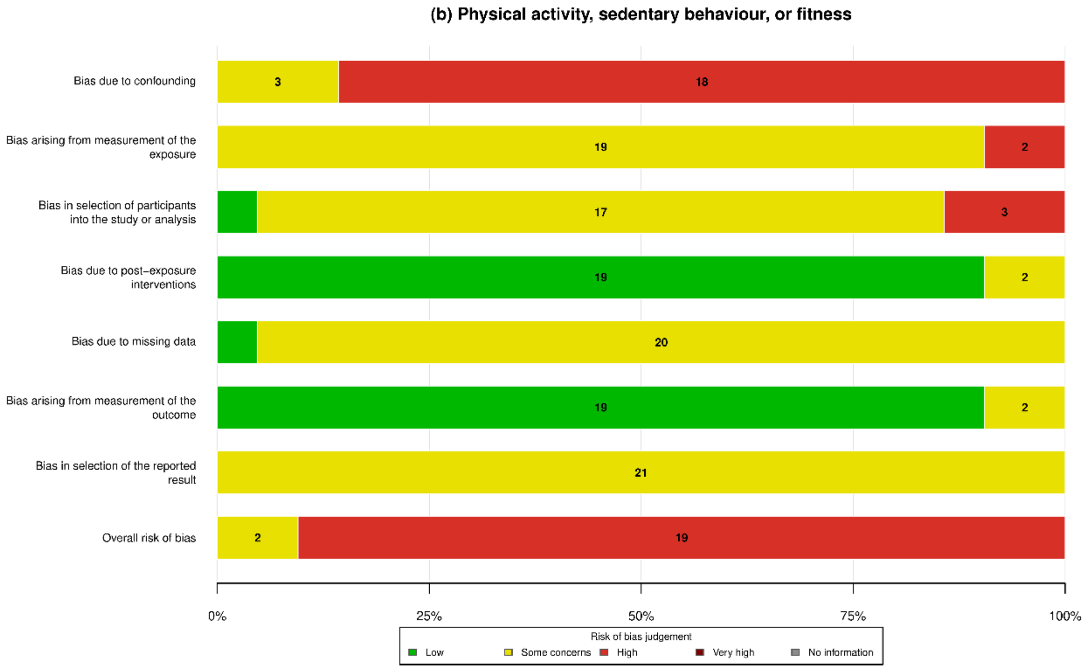

(a) Mediterranean diet adherence and (b) habitual physical activity, sedentary behaviour, or cardiorespiratory fitness.

Supplementary Table S1.

GRADE evidence profile for Mediterranean diet interventions and adherence, structured exercise or exercise-based cardiac rehabilitation, habitual physical activity, and joint lifestyle exposures in adults with established coronary heart disease.

Direction of effect: HR/RR <1.00 consistently favours the Mediterranean diet or more favourable physical-activity/lifestyle exposure; estimates representing different study designs or exposure contrasts are not pooled across estimands.

Evidence question	Design / tool	Outcome	Studies	Effect estimate or synthesis	Risk of bias	Inconsistency	Indirectness	Imprecision	Publication bias	Upgrading	Overall certainty
**A. Randomized intervention evidence**											
Mediterranean diet intervention	RCT / RoB 2	MACE	1	Intervention vs low-fat control: HR 0.72 (95% CI 0.54–0.96)	Not serious	Not assessable (1 study)	Not serious	Serious (a)	Not assessable	Not applicable	**Moderate** ⊕⊕⊕◯
Mediterranean diet intervention	RCT / RoB 2	Cardiovascular mortality	1	Intervention vs low-fat control: RR 0.55 (95% CI 0.27–1.13); 31 events	Not serious	Not assessable (1 study)	Not serious	Very serious (b)	Not assessable	Not applicable	**Low** ⊕⊕◯◯
Structured exercise / exercise-based cardiac rehabilitation	RCT / RoB 2	All-cause mortality	5	Narrative synthesis; no common intervention-effect estimand	Serious (c)	Serious (d)	Serious (d)	Very serious (e)	Not assessable	Not applicable	**Very low** ⊕◯◯◯
Structured exercise / exercise-based cardiac rehabilitation	RCT / RoB 2	Cardiovascular mortality	1	Narrative synthesis; sparse events	Serious (c)	Not assessable (1 study)	Serious (d)	Very serious (e)	Not assessable	Not applicable	**Very low** ⊕◯◯◯

Evidence question	Design / tool	Outcome	Studies	Effect estimate or synthesis	Risk of bias	Inconsistency	Indirectness	Imprecision	Publication bias	Upgrading	Overall certainty
Structured exercise / exercise-based cardiac rehabilitation	RCT / RoB 2	MACE / composite cardiovascular events	3	Narrative synthesis; intervention formats, comparators, and composite definitions differed	Serious (c)	Serious (d)	Serious (d, f)	Very serious (e)	Not assessable	Not applicable	**Very low** ⊕◯◯◯
**B. Comparative non-randomized intervention evidence**											
Structured exercise / exercise-based cardiac rehabilitation	NRSI / ROBINS-I V2	All-cause mortality	2	Narrative synthesis; no common intervention-effect estimand	Very serious (g)	Not assessable	Serious (h)	Very serious (e)	Not assessable	None	**Very low** ⊕◯◯◯
Structured exercise / exercise-based cardiac rehabilitation	NRSI / ROBINS-I V2	Cardiovascular mortality	3	Narrative synthesis; no common intervention-effect estimand	Very serious (g)	Not assessable	Serious (h)	Very serious (e)	Not assessable	None	**Very low** ⊕◯◯◯
Structured exercise / exercise-based cardiac rehabilitation	NRSI / ROBINS-I V2	MACE / composite cardiovascular events	2	Narrative synthesis; composite definitions and comparators differed	Very serious (g)	Not assessable	Serious (f, h)	Very serious (e)	Not assessable	None	**Very low** ⊕◯◯◯

Evidence question	Design / tool	Outcome	Studies	Effect estimate or synthesis	Risk of bias	Inconsistency	Indirectness	Imprecision	Publication bias	Upgrading	Overall certainty
**C. Observational exposure evidence**											
Mediterranean diet adherence	Cohort / ROBINS-E	All-cause mortality	4	Highest vs lowest: HR 0.74 (0.60–0.90), k=2, I^2=44%<br>Per 1-SD score increase: HR 0.91 (0.82–1.01), k=1<br>Per 1-point MDS increase: HR 0.96 (0.92–1.00), k=1<br>No pooled estimate across estimands.	Serious (i)	Not serious for pooled contrast; otherwise not assessable	Not serious	Serious for continuous contrasts (j)	Not assessable (<10 per estimand)	None	**Very low** ⊕◯◯◯
Mediterranean diet adherence	Cohort / ROBINS-E	Cardiovascular mortality	2	Highest vs lowest: HR 0.70 (0.43–1.14), k=1<br>Per 1-point MDS increase: HR 0.97 (0.92–1.03), k=1<br>No pooled estimate across estimands.	Serious (i)	Not assessable (single-study estimands)	Not serious	Serious (j)	Not assessable	None	**Very low** ⊕◯◯◯

Evidence question	Design / tool	Outcome	Studies	Effect estimate or synthesis	Risk of bias	Inconsistency	Indirectness	Imprecision	Publication bias	Upgrading	Overall certainty
Mediterranean diet adherence	Cohort / ROBINS-E	MACE	3	Highest vs lowest: HR 0.49 (0.29–0.82) and HR 0.78 (0.62–0.98) Per 1-point MDS increase: HR 0.95 (0.92–0.99) No pooled estimate because contrasts and composite definitions differed.	Serious (i)	Not assessable (non-comparable contrasts)	Serious (f, k)	Serious (j)	Not assessable	None	**Very low** ⊕◯◯◯

Evidence question	Design / tool	Outcome	Studies	Effect estimate or synthesis	Risk of bias	Inconsistency	Indirectness	Imprecision	Publication bias	Upgrading	Overall certainty
Habitual physical activity	Cohort / ROBINS-E	All-cause mortality	11	Maintained/continued vs persistently inactive: HR 0.64 (0.55–0.74), k=3, I²=29%<br>Meeting recommended target vs inactive: HR 0.69 (0.53–0.90), k=3, I²=90%<br>Higher vs lower categorical PA: HR 0.61 (0.47–0.79), k=3, I²=80%<br>Walking ≥100 vs <100 min/week below target: HR 0.84 (0.77–0.91), k=1<br>Per doubling exercise volume: HR 0.90 (0.87–0.93), k=1<br>No pooled estimate across estimands.	Serious (l)	Serious to very serious for two pooled estimands (m)	Not serious	Not serious	Not assessable (<10 per estimand)	None	**Very low** ⊕◯◯◯

Evidence question	Design / tool	Outcome	Studies	Effect estimate or synthesis	Risk of bias	Inconsistency	Indirectness	Imprecision	Publication bias	Upgrading	Overall certainty
Habitual physical activity	Cohort / ROBINS-E	Cardiovascular mortality	8	Maintained/continued vs persistently inactive: HR 0.42 (0.28–0.64), k=4, I^2=72%<br>Meeting recommended target vs inactive: HR 0.67 (0.47–0.95), k=2, I^2=91%<br>Higher vs lower categorical PA: HR 0.59 (0.46–0.75), k=1<br>Per doubling exercise volume: HR 0.92 (0.88–0.96), k=1<br>No pooled estimate across estimands.	Serious (l)	Serious to very serious for pooled estimands (m)	Not serious	Not serious	Not assessable (<10 per estimand)	None	**Very low** ⊕◯◯◯

Evidence question	Design / tool	Outcome	Studies	Effect estimate or synthesis	Risk of bias	Inconsistency	Indirectness	Imprecision	Publication bias	Upgrading	Overall certainty
Habitual physical activity	Cohort / ROBINS-E	MACE	6	Vigorous PA 1–2/week vs light: HR 0.82 (0.72–0.94)<br>MVPA continuation vs persistently inactive: HR 0.87 (0.79–0.96)<br>500–1000 MET-min/week vs inactive: HR 0.77 (0.43–1.38)<br>MVPA ≥94 vs <94 min/week: HR 0.77 (0.74–0.80)<br>Light pre-admission PA vs sedentary: HR 0.74 (0.58–0.95)<br>Walking ≥100 vs <100 min/week below target: HR 0.84 (0.71–0.99)<br>No pooled estimate across non-comparable contrasts.	Serious (l)	Not assessable (single-study estimands)	Serious (f, k)	Varied; serious for one contrast (j)	Not assessable	None	**Very low** ⊕◯◯◯

Evidence question	Design / tool	Outcome	Studies	Effect estimate or synthesis	Risk of bias	Inconsistency	Indirectness	Imprecision	Publication bias	Upgrading	Overall certainty
**D. Joint diet-and-physical-activity lifestyle evidence**											
Mediterranean lifestyle index incorporating diet, physical activity, rest, and social habits	Cohort / ROBINS-E	MACE	1	Higher vs lower joint lifestyle adherence: HR 0.28 (95% CI 0.09–0.86)	Serious (n)	Not assessable (1 study)	Serious (multicomponent exposure)	Serious	Not assessable	None (o)	**Very low** ⊕◯◯◯

GRADE approach and explanatory notes

Randomized evidence started at high certainty. Comparative non-randomized intervention and observational exposure evidence started at low certainty. Certainty was assessed separately by outcome, exposure/intervention, study design, and—where relevant—clinically distinct estimand. Evidence was downgraded for risk of bias, inconsistency, indirectness, imprecision, or suspected publication bias and considered for upgrading for a large effect, dose–response gradient, or plausible residual confounding that would reduce an observed association.

Effect-direction harmonization. All estimates were oriented so that HR/RR <1.00 favoured the more favourable lifestyle exposure or intervention: higher versus lower Mediterranean diet adherence; Mediterranean diet intervention versus control; higher, maintained, or increased activity versus lower, persistently inactive, or decreased activity; and structured exercise/cardiac rehabilitation versus usual care or control. For reverse comparisons, the harmonized HR was calculated as the reciprocal of the reported HR, with 95% CI limits calculated as 1/upper and 1/lower. Exposure and reference categories were checked against the original report by two reviewers. Directional harmonization did not change the underlying estimand, and clinically different contrasts remained separate.

For observational exposure evidence, the prespecified minimum confounder set comprised age, sex, smoking, socioeconomic status, CHD severity, baseline functional status or frailty, and other lifestyle behaviours; exposure-specific factors such as adiposity, comorbidity, and secondary-prevention treatment were additionally considered where relevant.

(a) Downgraded once for imprecision because the evidence was derived from one trial and the upper confidence limit approached no effect.

(b) Downgraded two levels for very serious imprecision because only 31 cardiovascular deaths occurred and the confidence interval included substantial benefit and possible harm.

(c) Two randomized exercise/cardiac rehabilitation reports had high overall risk of bias and three had some concerns.

(d) Exercise/cardiac rehabilitation formats, intensity, comparators, follow-up, and outcome definitions differed, preventing estimation of one common intervention effect.

(e) Event numbers were sparse, confidence intervals were wide or compatible effect estimates were unavailable.

(f) MACE was not treated as a uniform outcome. Exact study-specific composite definitions are reported in Supplementary Table S3; estimates with materially different definitions were not pooled.

(g) Three of four comparative non-randomized intervention studies had critical overall risk of bias and one had serious risk, principally because of confounding, time-zero or immortal-time concerns, and selection into the analysis.

(h) Comparators differed across non-randomized intervention studies, including no rehabilitation, PCI, and alternative management strategies.

(i) ROBINS-E identified residual confounding and, in some studies, self-reported dietary exposure measurement or selection concerns.

(j) Downgraded where confidence intervals included or closely approached no association, or where the evidence for a contrast came from a single study with limited information size.

(k) Mediterranean diet MACE estimates represented different adherence contrasts and non-uniform composite definitions; no cross-estimand synthesis was undertaken.

(l) Habitual physical-activity studies were predominantly at high overall risk of bias because of residual confounding, reverse causation, self-reported exposure measurement, and selection into repeated-measurement analyses.

(m) Heterogeneity was estimand-specific: I^2 was 29%, 90%, and 80% for the three pooled all-cause mortality contrasts and 72% and 91% for the two pooled cardiovascular mortality contrasts. Leave-one-out analyses preserved the direction and statistical significance of the pooled associations but did not consistently remove heterogeneity.

(n) The single joint Mediterranean lifestyle study had high overall risk of bias under ROBINS-E.

(o) The joint lifestyle evidence was not upgraded despite a large point estimate because it arose from one high-risk observational study with a wide confidence interval and a multicomponent exposure.

Publication bias was not formally assessed because fewer than 10 studies contributed to every compatible estimand-specific synthesis.

MACE, major adverse cardiovascular events; MDS, Mediterranean diet score; MET, metabolic equivalent of task; MVPA, moderate-to-vigorous physical activity; NRSI, non-randomized study of an intervention; PA, physical activity; RCT, randomized controlled trial; RoB 2, revised Cochrane Risk of Bias tool for randomized trials; ROBINS-E, Risk Of Bias In Non-randomized Studies—of Exposures; ROBINS-I V2, Risk Of Bias In Non-randomized Studies—of Interventions, Version 2.

.

Supplementary Table S2.

Study-specific definitions and components of major adverse cardiovascular events and other composite cardiovascular outcomes

This table includes every included report that used a named or author-constructed composite/aggregate cardiovascular outcome. Studies reporting only individual mortality or cardiovascular outcomes are not listed.

Study / exposure	Original outcome label	Exact components
Delgado-Lista et al. (2022)	Primary composite of major cardiovascular events	Myocardial infarction; coronary revascularization; ischemic stroke; documented peripheral artery disease; cardiovascular death.
Cangemi et al. (2023)	MACE	Cardiovascular death; myocardial infarction; stroke or transient ischemic attack.
Shikany et al. (2018)	Recurrent CHD event	First definite or probable nonfatal myocardial infarction or acute coronary heart disease death.
Díaz-Gutiérrez et al. (2026)	Adverse-episode composite	Acute myocardial infarction or new revascularization; new atrial fibrillation diagnosis; nonfatal stroke; all-cause mortality.
Lau et al. (2015)	MACE	Cardiovascular mortality; stroke (ischemic or hemorrhagic); acute coronary syndrome (unstable angina/NSTEMI or STEMI).
Kouvari et al. (2017)	Fatal/nonfatal recurrent ACS event	Vital status (cardiovascular or other death) and rehospitalization for acute coronary syndrome or other ischemia; reported WHO-ICD codes 410–414.9, 427.2, and 427.6.
Magnoni et al. (2020)	180-day MACE	Death (cause not restricted); nonfatal acute coronary syndrome (STEMI or NSTEMI); congestive heart failure; nonfatal stroke.
Stewart et al. (2016)	MACE	Cardiovascular death; nonfatal myocardial infarction; nonfatal stroke (time to first event).
Seron et al. (2024)	Cardiovascular events or mortality	Cardiovascular mortality (death due to stroke, myocardial infarction, or heart failure); new heart failure; or hospitalization for nonfatal stroke, nonfatal myocardial infarction, or revascularization surgery. The results also counted one pulmonary-sepsis death, although this was outside the stated cardiovascular-death definition.
Castro-Conde et al. (2021)	New cardiovascular event	Stroke; nonfatal myocardial infarction; new revascularization. Cardiovascular and all-cause mortality were recorded separately.
Tüner et al. (2025)	MACE	Death (cause not specified); myocardial infarction; hospitalization for unstable angina; stroke; unplanned revascularization.
He et al. (2020)	MACE	Cardiovascular death; nonfatal myocardial infarction; heart failure; stroke; cardiovascular-related rehospitalization (angina or chest distress with negative troponin T).

Chaves et al. (2019)	Morbidity aggregate	Cardiac emergency-department visit; cardiac hospital admission; nonfatal myocardial infarction; angina; PCI or CABG; heart failure. All-cause and cardiovascular mortality were separate outcomes.
Wu et al. (2024)	Endpoint events	Emergency visit or rehospitalization for angina; myocardial infarction; cardiovascular death.
Hiruma et al. (2024)	MACE after discharge	Cardiovascular death; stroke (brain hemorrhage or infarction); STEMI or NSTEMI; unstable angina; hospitalization for heart failure.
Guy et al. (2019)	MACE	In-stent restenosis; stent thrombosis; new significant coronary stenosis; myocardial infarction; heart failure; cardiac arrest; cardiac death.
Pavasini et al. (2024)	1-year primary composite	Death; myocardial infarction; stroke; ischemia-driven coronary revascularization.
Cho et al. (2025)	Primary cardiovascular composite	Nonfatal coronary event (hospitalization with ICD-10 I20–I25 plus PCI or CABG); nonfatal stroke (hospitalization with I60–I64 plus CT/MRI); cardiovascular mortality (I00–I99 cause of death).
Jin et al. (2022)	MACCE	Cardiovascular death; acute myocardial infarction; ischemic stroke; repeat revascularization (target or nontarget vessel); hospitalization for heart disease.
Lönn et al. (2025a)	MACE	Nonfatal cardiac event (acute MI, STEMI, or NSTEMI); stroke; heart failure; cardiovascular death.
Lönn et al. (2025b)	MACE	Nonfatal cardiac event (acute MI, STEMI, or NSTEMI); heart failure; stroke; cardiovascular death.
Yang et al. (2025)	Cardiovascular composite outcome	Cardiac death (death due to heart failure or CAD); heart-failure hospitalization; nonfatal myocardial infarction; PCI; CABG.
Biscaglia et al. (2020)	Primary composite	Cardiovascular death; myocardial infarction; stroke (first event).
Lönn et al. (2023)	First recurrent nonfatal CVD event	Hospital readmission for angina (I20), MI/reinfarction or MI complications (I21–I23), other ischemic heart disease (I24–I25), ischemic stroke (I63), or vascular dementia (F01).
Ek et al. (2019)	1-year CVD readmission	Hospital readmission for angina, heart failure, myocardial infarction, or stroke. Mortality was analyzed separately.
Stewart et al. (2017)	MACE	First occurrence of cardiovascular death, myocardial infarction, or stroke.

Abbreviations: ACS, acute coronary syndrome; AF, atrial fibrillation; CABG, coronary artery bypass grafting; CAD, coronary artery disease; CHD, coronary heart disease; CR, cardiac rehabilitation; CV, cardiovascular; HF, heart failure; ICD, International Classification of Diseases; IHD, ischemic heart disease; MACE, major adverse cardiovascular events; MACCE, major adverse cardiac and cerebrovascular events; MI, myocardial infarction; MINOCA, myocardial infarction with nonobstructive coronary arteries; MVPA, moderate-to-vigorous physical activity; NSTEMI, non-ST-elevation myocardial infarction; PA, physical activity; PCI, percutaneous coronary

intervention; RCT, randomized controlled trial; STEMI, ST-elevation myocardial infarction; TIA, transient ischemic attack.

Supplementary Table S3.

Report-to-parent-cohort mapping and management of overlapping populations

ID	Report	Parent cohort / data source	Report N	Overlap classification	Contribution to the review and synthesis decision	Participant-counting rule
C01	Delgado-Lista et al. (2022)	CORDIOPREV randomized trial	1,002	No known overlap	Mediterranean-diet intervention; MACE and cardiovascular mortality	Count once (N=1,002)
C02	Cangemi et al. (2023)	EVA study (Italian ischemic-heart-disease cohort)	503	No known overlap	Mediterranean-diet adherence; MACE; observational estimate	Count once (N=503)
C03	Shikany et al. (2018)	REGARDS cohort	3,562	No known overlap	Mediterranean-diet adherence; categorical all-cause mortality and MACE estimates	Count once (N=3,562)
C04	Díaz-Gutiérrez et al. (2026)	MEDLIFE pilot cohort	115	No known overlap	Joint Mediterranean-lifestyle index; MACE; displayed separately	Count once (N=115)
C05	Lau et al. (2015)	Hong Kong stable-CAD cohort	274	No known overlap	Mediterranean-diet adherence; narrative/non-pooled evidence	Count once (N=274)
C06	Kouvari et al. (2017)	Hellenic Heart Failure Study	690	No known overlap	Mediterranean-diet adherence; cardiovascular prognosis; narrative evidence	Count once (N=690)
C07	Magnoni et al. (2020)	FAMI first-STEMI study	533	No known overlap	Mediterranean-diet adherence; short-term cardiovascular outcomes; narrative evidence	Count once (N=533)
**C08†**	Liang et al. (2022)	NHANES (1999–2010 cycles)	2,052	Partial source overlap with Pan et al.	Highest-versus-lowest adherence; included in categorical all-cause mortality synthesis and displayed separately for cardiovascular mortality	NHANES counted once at source level (N=3,088; conservative rule)

ID	Report	Parent cohort / data source	Report N	Overlap classification	Contribution to the review and synthesis decision	Participant-counting rule
**C08†**	Pan et al. (2025)	NHANES (2005–2018 cycles)	3,088	Partial source overlap with Liang et al.	Per 1-SD diet-score estimate; retained as a separate estimand and never pooled with Liang et al.	NHANES counted once at source level (N=3,088; conservative rule)
**C09†**	Stewart et al. (2016)	STABILITY trial cohort	15,482	Near-identical population to Stewart et al. (2017)	Mediterranean-diet adherence; diet-specific mortality and MACE estimands	STABILITY counted once (N=15,486)
C10	Seron et al. (2024)	Chilean hybrid cardiac-rehabilitation randomized trial	191	No known overlap	Structured exercise/cardiac rehabilitation; randomized intervention evidence	Count once (N=191)
C11	Castro-Conde et al. (2021)	MxM (Más por Menos) randomized trial	497	No known overlap	Intensive versus standard cardiac rehabilitation; randomized intervention evidence	Count once (N=497)
C12	Pavasini et al. (2024)	FIRE randomized-trial cohort	1,445	No known overlap	Pre-admission physical activity as an observational exposure; MACE displayed separately	Count once (N=1,445)
C13	Tüner et al. (2025)	Turkish ACS cardiac-rehabilitation randomized trial	340	No known overlap	Exercise-based cardiac rehabilitation; randomized intervention evidence	Count once (N=340)
C14	He et al. (2020)	Chinese MINOCA cardiac-rehabilitation trial	524	No known overlap	Exercise-based cardiac rehabilitation; intervention evidence	Count once (N=524)
C15	Chaves et al. (2019)	Brazilian wait-list cardiac-rehabilitation trial	115	No known overlap	Comprehensive/exercise-only cardiac rehabilitation; randomized evidence	Count once (N=115)
C16	Wu et al. (2024)	Chinese stable-angina comparative cohort	100	No known overlap	Exercise rehabilitation plus OMT versus PCI plus OMT; non-randomized intervention evidence	Count once (N=100)

ID	Report	Parent cohort / data source	Report N	Overlap classification	Contribution to the review and synthesis decision	Participant-counting rule
C17	Taylor et al. (2016)	UK community exercise-rehabilitation cohort	670	No known overlap	Post-rehabilitation cardiorespiratory fitness; all-cause mortality	Count once (N=670)
C18	Hiruma et al. (2024)	Japanese AMI cardiac-rehabilitation cohort	610	No known overlap	Comprehensive cardiac rehabilitation; non-randomized intervention evidence	Count once (N=610)
C19	Lin et al. (2016)	Taiwan National Health Insurance Research Database	6,713	No known overlap	Phase-I cardiac rehabilitation; sex-specific prognosis; narrative evidence	Count once (N=6,713)
C20	Guy et al. (2019)	French multicentre post-PCI sports cohort	108	No known overlap	Intensive/competitive versus moderate sport; MACE; narrative evidence	Count once (N=108)
**C21†**	Ek et al. (2019)	SWEDEHEART/SEPHIA (2004–2014)	30,614	Potential participant overlap with Lönn et al. (2023)	Post-MI physical-activity category; included in categorical all-cause mortality synthesis	SWEDEHEART counted once at source level (N=47,153; conservative rule)
C22	Li et al. (2023)	Chinese nationwide MI-survivor cohort	20,653	No known overlap	Higher-versus-lower physical activity; all-cause mortality synthesis	Count once (N=20,653)
C23	Buckley et al. (2022)	TriNetX global research network	18,383	No known overlap	Exercise-based cardiac rehabilitation versus PCI; non-randomized intervention evidence	Count once (N=18,383)
C24	Lee et al. (2016)	Asan-MAIN Revascularization Registry	3,040	No known overlap	Cardiac-rehabilitation participation in left-main CAD; non-randomized intervention evidence	Count once (N=3,040)
**C21†**	Lönn et al. (2023)	SWEDEHEART-CR (2005–2019)	47,153	Potential participant overlap with Ek et al. (2019)	Physical-activity change; recurrent nonfatal cardiovascular events; not pooled with Ek et al.	SWEDEHEART counted once at source level (N=47,153; conservative rule)

ID	Report	Parent cohort / data source	Report N	Overlap classification	Contribution to the review and synthesis decision	Participant-counting rule
C25	Yang et al. (2025)	UK Biobank	19,074	No known overlap	Walking/physical-activity estimands; all-cause mortality and MACE displayed separately	Count once (N=19,074)
**C26†**	Lönn et al. (2025a)	Australian 45 and Up Study	40,156	Same baseline cohort as Freene et al.; parent cohort of Lönn et al. (2025b)	Data-derived MVPA threshold; MACE displayed separately	45 and Up Study counted once (N=40,156)
**C26†**	Lönn et al. (2025b)	Australian 45 and Up Study repeated-assessment subset	9,430	Nested subset of the 40,156-participant cohort	Longitudinal MVPA/sedentary-behaviour change; retained as narrative evidence, not as an additional independent primary estimate	45 and Up Study counted once (N=40,156)
**C27†**	Jae et al. (2025)	Korean NHIS ACS cohort (2010–2017)	30,840	Identical cohort to Cho et al. (2025)	Physical-activity continuation/change; selected for all-cause and cardiovascular mortality	Korean NHIS ACS cohort counted once (N=30,840)
**C27†**	Cho et al. (2025)	Korean NHIS ACS cohort (2010–2017)	30,840	Identical cohort to Jae et al. (2025)	Physical-activity continuation/change; selected for MACE; mortality estimate not additionally synthesized	Korean NHIS ACS cohort counted once (N=30,840)
**C26†**	Freene et al. (2024)	Australian 45 and Up Study	40,156	Same baseline cohort as Lönn et al. (2025a)	Recommended-target versus inactivity; selected for all-cause and cardiovascular mortality	45 and Up Study counted once (N=40,156)
C28	Al-Shaar et al. (2020)	Health Professionals Follow-up Study	1,651	No known overlap	Maintained/increased physical activity after MI; mortality syntheses	Count once (N=1,651)
C29	Gorczyca et al. (2017)	Women's Health Initiative Observational Study	856	No known overlap	Maintained/increased physical activity after MI; mortality syntheses	Count once (N=856)
C30	Biscaglia et al. (2020)	CLARIFY registry	32,370	No known overlap	Physical-activity intensity; MACE displayed separately	Count once (N=32,370)

ID	Report	Parent cohort / data source	Report N	Overlap classification	Contribution to the review and synthesis decision	Participant-counting rule
C31	Lahtinen et al. (2018)	ARTEMIS cohort	1,746	No known overlap	Longitudinal physical-activity category; cardiovascular mortality synthesis	Count once (N=1,746)
C32	Jin et al. (2022)	South Korean single-centre AF–CAD registry	551	No known overlap	Recommended physical-activity target; all-cause mortality and MACE estimands	Count once (N=551)
C33	Moholdt et al. (2017)	HUNT Study	6,493	No known overlap	Recommended physical-activity target; mortality syntheses	Count once (N=6,493)
**C09†**	Stewart et al. (2017)	STABILITY trial cohort	15,486	Near-identical population to Stewart et al. (2016)	Exercise volume per doubling; physical-activity mortality estimands; separate review question	STABILITY counted once (N=15,486)
C34	Bouisset et al. (2020)	GENES cohort	822	No known overlap	Higher-versus-lower physical activity; all-cause mortality synthesis	Count once (N=822)

† Overlapping cluster.

Abbreviations: ACS, acute coronary syndrome; AF, atrial fibrillation; AMI, acute myocardial infarction; CAD, coronary artery disease; CR, cardiac rehabilitation; MACE, major adverse cardiovascular events; MI, myocardial infarction; MINOCA, myocardial infarction with non-obstructive coronary arteries; MVPA, moderate-to-vigorous physical activity; NHANES, National Health and Nutrition Examination Survey; NHIS, National Health Insurance Service; OMT, optimal medical therapy; PCI, percutaneous coronary intervention; SD, standard deviation; STEMI, ST-segment elevation myocardial infarction.

Supplementary Table S4.

Study-level assessment methods and contribution to outcome-specific quantitative or narrative synthesis

Interpretation. The review included 40 eligible reports representing 34 parent cohorts or data sources. Eligibility for the systematic review did not automatically imply eligibility for quantitative synthesis. Pooling required the same evidence stratum and sufficiently comparable target estimand, intervention or exposure contrast, outcome definition, and effect measure. ‘Displayed separately’ denotes an unpooled forest estimate, whereas ‘narrative synthesis only’ denotes an eligible report without a compatible quantitative estimate. Mediterranean diet scores were not treated as interchangeable. Structured exercise or cardiac rehabilitation was separated from observational habitual physical activity.

Report and study design	Evidence question / exposure class	Assessment instrument or intervention protocol; scale, metric, and operational contrast	Eligible outcome(s)	Synthesis contribution and location	Reason for non-pooling or handling decision
**A. Mediterranean diet intervention, adherence, and combined lifestyle indices**					
**C01**<br>**Delgado-Lista et al. (2022)**<br>*Randomized controlled trial*	Mediterranean diet intervention effect	Assigned intervention. Dietitian-delivered Mediterranean diet versus an active low-fat diet; 14-item Mediterranean Diet Adherence Screener (0–14) used for adherence monitoring; median intervention duration 7 years.	Cardiovascular mortality; expanded MACE	**Displayed separately; not pooled**<br>Figure 4 (intervention stratum); Table S1	Single randomized trial. Intervention effects were kept separate from observational adherence associations; cardiovascular deaths were sparse and the composite endpoint was expanded MACE.
**C02**<br>**Cangemi et al. (2023)**<br>*Prospective cohort*	Mediterranean diet adherence association	Self-reported validated 14-item Mediterranean diet questionnaire, completed with dietitian support; score 0–14; low ≤5, moderate 6–9, high ≥10; moderate/high versus low adherence.	MACE variant	**Displayed separately; not pooled**<br>Figure 4 (adherence stratum)	Observational association; composite included transient ischemic attack and was not equivalent to recurrent CHD or core MACE. It was also kept separate from the randomized intervention effect.
**C03**<br>**Shikany et al. (2018)**<br>*Prospective cohort*	Mediterranean diet adherence association	Self-administered Block 98 semiquantitative FFQ assessing usual diet in the previous year; 9-component MDS (0–9); categories 0–3, 4–5, and 6–9; highest versus lowest.	All-cause mortality; recurrent CHD event	**Pooled for all-cause mortality; recurrent CHD displayed separately**<br>Figure 2, 1.1;<br>Figure 4	The all-cause mortality estimate was comparable with Liang et al. and was pooled. The recurrent CHD composite (nonfatal MI or acute CHD death) was not classified as core MACE and was not pooled with other composites.

Report and study design	Evidence question / exposure class	Assessment instrument or intervention protocol; scale, metric, and operational contrast	Eligible outcome(s)	Synthesis contribution and location	Reason for non-pooling or handling decision
**C04**<br>**Díaz-Gutiérrez et al. (2026)**<br>*Prospective cohort*	Joint diet-and-physical-activity lifestyle evidence	Self-administered 30-item MEDLIFE index: Mediterranean food 0–15, dietary habits/cooking 0–7, and physical activity/rest/social habits 0–8; total 0–30; sex-specific tertiles, highest versus lowest.	Mixed adverse-episode composite	**Displayed separately; not pooled**<br>Figure 4 (joint-lifestyle stratum); Section 3.9	MEDLIFE jointly incorporated diet, physical activity, rest, and social habits. Its composite included MI/revascularization, incident AF, stroke, and all-cause death; it was therefore a separate review question.
**C05**<br>**Lau et al. (2015)**<br>*Prospective cohort*	Mediterranean diet adherence association	Validated Chinese FFQ; 8-category MDS (0–8); higher versus lower score and continuous per 1-point estimates.	MACE; all-cause and cardiovascular mortality; stroke	**Narrative synthesis only**<br>Narrative evidence; Tables S2 and S4	An adjusted HR was available for stroke, but not for the prespecified MACE or mortality contrasts. The reported composite also combined stroke, ACS, and cardiovascular death; no compatible target-outcome HR was available for pooling.
**C06**<br>**Kouvari et al. (2017)**<br>*Prospective cohort*	Mediterranean diet adherence association	Nutritionist-administered validated 75-item semiquantitative FFQ; MedDietScore (0–55); tertiles 0–16, 17–20, and 21–55 and per 1-point increase; heart-failure phenotype analyses.	Fatal/nonfatal recurrent ACS	**Narrative synthesis only**<br>Section 3.9; Tables S2 and S4	The study reported odds ratios for recurrent ACS rather than time-to-event HRs. Recurrent ACS was not considered equivalent to MACE, and estimates varied by heart-failure phenotype and model adjustment.
**C07**<br>**Magnoni et al. (2020)**<br>*Prospective cohort*	Mediterranean diet adherence association	Interviewer-administered FFQ assessing pre-STEMI diet; FAMI Mediterranean Diet Score adapted from MedDietScore, observed range 12–37; score quintiles and higher versus lower fruit/vegetable intake.	180-day all-cause mortality; expanded MACE	**Narrative synthesis only**<br>Narrative evidence; Tables S2 and S4	Only short-term event proportions were reported, without a compatible adjusted HR. The composite included all-cause death, ACS, heart failure, and stroke and was not comparable with core MACE.

Report and study design	Evidence question / exposure class	Assessment instrument or intervention protocol; scale, metric, and operational contrast	Eligible outcome(s)	Synthesis contribution and location	Reason for non-pooling or handling decision
**C08†**<br>**Liang et al. (2022)**<br>*Prospective cohort*	Mediterranean diet adherence association	NHANES dietary-recall data linked to the USDA Food Patterns Equivalents Database; aMED score (0–9); categories 0–2, 3, and 4–9; highest versus lowest.	All-cause and cardiovascular mortality	**Pooled for all-cause mortality; cardiovascular mortality displayed separately**<br>Figure 2, 1.1;<br>Figure 3, 1.1	The categorical all-cause mortality estimate was pooled with Shikany et al. Cardiovascular mortality was a single-study categorical estimand and was not pooled with Stewart's continuous score estimate.
**C08†**<br>**Pan et al. (2025)**<br>*Prospective cohort*	Mediterranean diet adherence association	Two computer-assisted 24-hour dietary recalls; 9-component Mediterranean Diet Score (0–9); quartiles and per 1-SD increase; the current synthesis uses the per 1-SD estimate.	All-cause mortality	**Displayed separately; not pooled**<br>Figure 2, 1.2	The standardized continuous contrast was not equivalent to highest-versus-lowest adherence. The NHANES source partially overlapped with Liang et al.; the estimates were kept in separate estimand strata and never pooled together.
**C09†**<br>**Stewart et al. (2016)**<br>*Prospective trial-cohort analysis*	Mediterranean diet adherence association	Short self-reported lifestyle FFQ; 7-component MDS (0–28); categories ≤12, 13–14, and ≥15; current estimates are per 1-point increase among participants with MDS >12.	All-cause mortality; cardiovascular mortality; MACE	**Displayed separately; not pooled**<br>Figure 2, 1.3;<br>Figure 3, 1.2; Figure 4	The continuous score increment was not equivalent to categorical adherence contrasts. The report shared the STABILITY parent cohort with Stewart et al. (2017), which addressed a different physical-activity question.
**B. Structured exercise, cardiac rehabilitation, fitness, and contextual intervention evidence**					
**C10**<br>**Seron et al. (2024)**<br>*Randomized controlled trial*	Structured exercise/cardiac rehabilitation intervention effect	Assigned intervention. Hybrid CR comprised 10 supervised sessions over 4–6 weeks plus home support to week 12; standard CR comprised 18–22 supervised sessions over 8–12 weeks; aerobic/resistance exercise, Borg 12–13.	All-cause mortality; broad cardiovascular event/mortality composite	**Narrative synthesis only**<br>Structured-CR narrative; Table S1	Active-intervention comparator, sparse events, and a broad composite. The reported RR/limited survival information did not form a common randomized time-to-event estimand with the other CR trials.

Report and study design	Evidence question / exposure class	Assessment instrument or intervention protocol; scale, metric, and operational contrast	Eligible outcome(s)	Synthesis contribution and location	Reason for non-pooling or handling decision
**C11**<br>**Castro-Conde et al. (2021)**<br>*Randomized controlled trial*	Structured cardiac rehabilitation intervention effect	Assigned intervention. Both arms used the same supervised aerobic/resistance CR program; intensive CR comprised 10 consecutive daily sessions over 2 weeks plus boosters at months 3, 6, and 9; standard CR comprised 24 sessions over 8 weeks.	All-cause and cardiovascular mortality; new cardiovascular events	**Narrative synthesis only**<br>Section 3.9; Table S1	Both groups received active multicomponent rehabilitation. Sparse events and the active-comparator estimand were not aligned with usual-care/no-CR trials or habitual physical-activity associations.
**C13**<br>**Tüner et al. (2025)**<br>*Randomized controlled trial*	Structured cardiac rehabilitation intervention effect	Assigned intervention. Twelve-week outpatient multidisciplinary CR versus standard recommendations; aerobic exercise 3 times/week for 30–45 min at 60%–70% of peak heart rate and resistance exercise twice/week. Accelerometry and logs assessed adherence, not exposure.	All-cause mortality; readmission; revascularization; expanded MACE	**Narrative synthesis only**<br>Structured-CR narrative; Table S1	No deaths occurred. The time-to-event model primarily concerned revascularization/adherence, and the expanded composite and effect scale were not sufficiently comparable with other intervention studies.
**C14**<br>**He et al. (2020)**<br>*Randomized controlled trial*	Structured cardiac rehabilitation intervention effect	Assigned intervention. In-hospital supervised treadmill/cycle exercise for 20–30 min 3 times/week, followed by 3 years of home moderate continuous training for 47 min 3 times/week at 65%–75% of maximum heart rate, monitored by heart-rate band and WeChat.	All-cause mortality; expanded MACE	**Narrative synthesis only**<br>Structured-CR narrative; Table S1	Single MINOCA intervention study with a distinct population and an expanded MACE definition including heart failure and cardiovascular rehospitalization. It was not pooled with observational activity studies or dissimilar CR trials.

Report and study design	Evidence question / exposure class	Assessment instrument or intervention protocol; scale, metric, and operational contrast	Eligible outcome(s)	Synthesis contribution and location	Reason for non-pooling or handling decision
**C15**<br>**Chaves et al. (2019)**<br>*Wait-list randomized crossover trial*	Structured cardiac rehabilitation intervention effect	Assigned comprehensive CR, exercise-only CR, or wait-list. The 6-month program included 36 supervised 1-hour exercise sessions; the comprehensive arm added 24 education sessions; wait-list participants later crossed over.	All-cause mortality; morbidity aggregate; hospitalization	**Narrative synthesis only**<br>Structured-CR narrative; Table S1	Wait-list crossover design, sparse events, and a broad morbidity aggregate including health-care use and revascularization. No common time-to-event intervention estimand was available.
**C16**<br>**Wu et al. (2024)**<br>*Non-randomized comparative cohort*	Comparative non-randomized intervention effect	Nonrandomized treatment strategy: OMT plus home exercise rehabilitation versus OMT plus PCI. Home moderate exercise lasted 30 min 3–4 times/week for 1 year, with heart rate controlled at the anaerobic threshold and regular supervision.	Cardiovascular death; MI; angina/emergency visit or rehospitalization	**Narrative synthesis only**<br>Comparative-NRSI narrative; Table S1	PCI was an active therapeutic comparator rather than usual care or lower exercise exposure. The study addressed a distinct treatment-strategy effect and was not pooled with CR-vs-no-CR or habitual-activity estimates.
**C17**<br>**Taylor et al. (2016)**<br>*Retrospective cohort*	Cardiorespiratory-fitness association (separate stratum)	Objective post-rehabilitation submaximal treadmill/cycle test; fitness estimated in METs at 85% of age-predicted maximum heart rate or Borg 16–17; low <6 METs in men/<5 in women and high ≥8 in men/≥7 in women.	All-cause mortality	**Narrative synthesis only**<br>Fitness narrative; Table S1	Cardiorespiratory fitness was treated as a phenotype/prognostic marker rather than habitual physical activity or an assigned exercise intervention. It was retained only as a separate exposure stratum.
**C18**<br>**Hiruma et al. (2024)**<br>*Retrospective cohort*	Comparative non-randomized cardiac-rehabilitation effect	Outpatient comprehensive CR versus inpatient-only CR. Outpatient CR continued for up to 150 days with supervised treadmill/cycle exercise 1–3 times/week for 30 min at anaerobic-threshold workload/Borg 11–13, optional resistance training, and multidisciplinary counseling.	MACE; cardiovascular mortality; heart-failure hospitalization	**Narrative synthesis only**<br>Comparative-NRSI narrative; Table S1	Non-randomized participation effect with substantial confounding risk and an expanded MACE definition. No sufficiently aligned non-randomized intervention estimand was available for pooling.

Report and study design	Evidence question / exposure class	Assessment instrument or intervention protocol; scale, metric, and operational contrast	Eligible outcome(s)	Synthesis contribution and location	Reason for non-pooling or handling decision
**C19**<br>**Lin et al. (2016)**<br>*Retrospective claims cohort*	Sex-specific prognosis during Phase-I rehabilitation (contextual evidence)	NHIRD procedure-coded Phase-I inpatient CR; therapist-guided monitored exercise was prescribed according to clinical condition. All participants received CR; the comparison was women versus men rather than rehabilitation dose.	All-cause, in-hospital, and 30-day mortality	**Narrative synthesis only**<br>Separate contextual narrative	The comparison was a sex contrast rather than an exercise or rehabilitation contrast. It was retained for contextual evidence but did not enter any intervention or exposure meta-analysis.
**C23**<br>**Buckley et al. (2022)**<br>*Retrospective electronic-health-record cohort*	Comparative non-randomized intervention effect	Electronic-health-record codes for exercise counseling/outpatient CR within 6 months of chronic coronary syndrome diagnosis; exercise-based CR versus PCI and CR plus PCI versus CR. Actual exercise dose was unavailable.	All-cause mortality; readmission; nonfatal cardiovascular events; heart failure	**Narrative synthesis only**<br>Comparative-NRSI narrative; Table S1	PCI was an active therapeutic comparator and the propensity-matched analysis reported odds ratios rather than a common HR intervention estimand. It was not pooled with usual-care/no-CR comparisons.
**C24**<br>**Lee et al. (2016)**<br>*Registry cohort*	Comparative non-randomized cardiac-rehabilitation effect	Registry-defined Phase-II CR participation as ≥1 outpatient session within 3 months; standardized individualized multidisciplinary CR; median 12 sessions (IQR 7–15); participation versus no participation.	All-cause and cardiovascular mortality; MI; stroke	**Narrative synthesis only**<br>Comparative-NRSI narrative; Table S1	Non-randomized participation effect in a distinct left-main CAD population, with substantial residual confounding. No sufficiently aligned non-randomized intervention stratum was available for pooling.
**C. Observational habitual physical activity, sedentary behaviour, and sport-intensity evidence**					
**C12**<br>**Pavasini et al. (2024)**<br>*Observational analysis within a randomized-trial cohort*	Habitual/pre-admission physical-activity association	Patient/relative interview during the index admission; pre-admission activity classified as sedentary, light most weeks, or vigorous for ≥20 min 1–2 or ≥3 times/week; the displayed estimate is light versus sedentary.	MACE	**Displayed separately; not pooled**<br>Figure 7, 1.5	This was an observational exposure analysis within a randomized-trial cohort, not a randomized exercise effect. The pre-admission contrast was unique and was not equivalent to the other MACE activity contrasts.

Report and study design	Evidence question / exposure class	Assessment instrument or intervention protocol; scale, metric, and operational contrast	Eligible outcome(s)	Synthesis contribution and location	Reason for non-pooling or handling decision
**C20**<br>**Guy et al. (2019)**<br>*Retrospective multicentre cohort*	Post-PCI sport-intensity association (separate stratum)	Self-reported questionnaire on post-PCI sport type, regularity, frequency, duration, and intensity; inclusion required ≥4 h/week. Moderate leisure sport below the ventilatory threshold was compared with intensive leisure sport above the threshold and competitive sport.	MACE; recurrent cardiovascular events; cardiac death	**Narrative synthesis only**<br>Sport-intensity narrative; Tables S2 and S4	Active sport-intensity comparator, highly selected male post-PCI population, small event count, and an expanded coronary composite. The contrast was not equivalent to higher-vs-lower habitual activity or inactivity.
**C21†**<br>**Ek et al. (2019)**<br>*Prospective registry cohort*	Habitual physical-activity association	Self-reported 7-day recall at 6–10 weeks after MI; sessions lasting ≥30 min at an intensity of at least fast walking. Low 0–1, medium 2–4, and high 5–7 sessions/week; higher versus low activity.	All-cause mortality	**Pooled (estimand-specific)**<br>Figure 5, 1.3	The categorical all-cause mortality contrast was sufficiently comparable with Bouisset and Li. Potential SWEDEHEART overlap with Lönn et al. (2023) was handled by outcome-specific estimate selection.
**C22**<br>**Li et al. (2023)**<br>*Prospective cohort*	Habitual physical-activity association	Standardized in-person questionnaire covering leisure, household, occupational, and transport activity in the previous year; total MET-min/week; insufficient <3000, moderate 3000–4500, and high >4500; sufficient versus insufficient activity.	All-cause and cardiovascular mortality	**Pooled for all-cause mortality; cardiovascular mortality displayed separately**<br>Figure 5, 1.3;<br>Figure 6, 1.3	The all-cause mortality contrast was pooled with comparable categorical estimates. Cardiovascular mortality was a single-study categorical estimand and was not pooled across different activity contrasts.

Report and study design	Evidence question / exposure class	Assessment instrument or intervention protocol; scale, metric, and operational contrast	Eligible outcome(s)	Synthesis contribution and location	Reason for non-pooling or handling decision
**C21†**<br>**Lönn et al. (2023)**<br>*Prospective registry cohort*	Longitudinal physical-activity association	Validated self-reported single-item measure at approximately 2 and 12 months after MI: 0–7 sessions/week of moderate/vigorous activity lasting ≥30 min. Inactive 0–1 versus active ≥2 sessions; remaining inactive, increasing, decreasing, or remaining active.	Broad recurrent nonfatal cardiovascular composite	**Narrative synthesis only**<br>PA-change narrative; Tables S2–S4	The composite included angina, broad ischemic heart disease, ischemic stroke, and vascular dementia and was not classified as MACE. Potential SWEDEHEART overlap with Ek et al. was also considered.
**C25**<br>**Yang et al. (2025)**<br>*Prospective cohort*	Habitual walking association	Self-reported short-form IPAQ at UK Biobank baseline; walking, moderate, and vigorous activity converted to MET-min/week. Among participants below the MVPA recommendation, walking ≥100 versus <100 min/week.	All-cause mortality; MACE	**Displayed separately; not pooled**<br>Figure 5, 1.4;<br>Figure 7, 1.6	The restricted-population walking threshold was not equivalent to meeting the overall activity target, maintained activity, or general higher-vs-lower activity contrasts.
**C26†**<br>**Lönn et al. (2025a)**<br>*Prospective cohort*	Habitual physical-activity/sedentary-behaviour association	Self-reported Active Australia Survey; total MVPA = walking + moderate + 2×vigorous minutes/week. Data-derived survival-tree threshold for MACE: ≥94 versus <94 min/week; sitting time was also self-reported.	MACE	**Displayed separately; not pooled**<br>Figure 7, 1.4	Data-derived activity threshold and an expanded MACE definition including heart failure. It shared the 45 and Up parent cohort with Freene et al.; this report was selected for MACE only.
**C26†**<br>**Lönn et al. (2025b)**<br>*Prospective repeated-assessment cohort*	Longitudinal physical-activity/sedentary-behaviour association	Repeated self-reported Active Australia Survey and sitting questions; total MVPA = walking + moderate + 2×vigorous minutes/week. Outcome- and sex-specific thresholds at both waves defined remaining high, increasing, decreasing, and remaining low patterns.	MACE; cardiovascular mortality	**Narrative synthesis only**<br>Longitudinal-change narrative; Table S3	Repeated-assessment subset nested within the 45 and Up cohort. Its longitudinal change estimand was distinct, and it was retained narratively to avoid dependent use alongside Freene and Lönn et al. (2025a).

Report and study design	Evidence question / exposure class	Assessment instrument or intervention protocol; scale, metric, and operational contrast	Eligible outcome(s)	Synthesis contribution and location	Reason for non-pooling or handling decision
**C27†**<br>**Jae et al. (2025)**<br>*Population-based cohort*	Longitudinal physical-activity association	Modified IPAQ at health examinations before and after ACS; self-reported frequency and duration converted to MET-min/week. MVPA criterion: ≥5 days/week moderate or ≥3 days/week vigorous activity; initiation, cessation, or continuation versus persistent inactivity.	All-cause and cardiovascular mortality	**Pooled (estimand-specific)**<br>Figure 5, 1.1;<br>Figure 6, 1.1	The longitudinal mortality contrasts were comparable with the corresponding estimand strata. This report was selected for mortality from the Korean NHIS ACS cohort; Cho et al. was selected for MACE.
**C27†**<br>**Cho et al. (2025)**<br>*Population-based cohort*	Longitudinal physical-activity association	Modified IPAQ at health examinations within 3 years before and 2 years after ACS; light, moderate, and vigorous activity converted to MET-min/week. MVPA was ≥1 day/week of moderate activity for ≥30 min or vigorous activity for ≥20 min; continuation versus persistent inactivity.	MACE	**Displayed separately; not pooled**<br>Figure 7, 1.2	Same Korean NHIS ACS cohort as Jae et al.; this report was selected only for MACE. Its longitudinal contrast and composite definition were not equivalent to the other five MACE estimates.
**C26†**<br>**Freene et al. (2024)**<br>*Prospective cohort*	Habitual physical-activity association	Self-reported Active Australia Survey; total MVPA = walking + moderate + 2×vigorous minutes/week. Categories 0, 1–149, 150–300, and >300 min/week; the synthesis contrast is meeting the target versus inactivity.	All-cause and cardiovascular mortality	**Pooled (estimand-specific)**<br>Figure 5, 1.2;<br>Figure 6, 1.2	The target-versus-inactivity contrast was pooled only with comparable mortality estimates. This report was selected for mortality from the 45 and Up parent cohort.

Report and study design	Evidence question / exposure class	Assessment instrument or intervention protocol; scale, metric, and operational contrast	Eligible outcome(s)	Synthesis contribution and location	Reason for non-pooling or handling decision
**C28**<br>**Al-Shaar et al. (2020)**<br>*Prospective cohort*	Longitudinal physical-activity association	Validated biennial self-administered HPFS physical-activity questionnaire; activity-specific duration converted to MET-h/week. Change threshold 7.5 MET-h/week; maintained low, decreased, increased, or maintained high activity after MI.	All-cause and cardiovascular mortality	**Pooled (estimand-specific)**<br>Figure 5, 1.1;<br>Figure 6, 1.1	The longitudinal activity contrast and mortality outcomes were sufficiently comparable with the corresponding maintained/continued-activity strata.
**C29**<br>**Gorczyca et al. (2017)**<br>*Prospective cohort*	Longitudinal physical-activity association	Validated WHI recreational-activity questionnaire and sitting-time question before and after MI; activity converted to MET-h/week. High activity ≥7.5 MET-h/week and high sitting ≥8 h/day; maintained low, decreased, increased, or maintained high.	All-cause and cardiovascular mortality	**Pooled (estimand-specific)**<br>Figure 5, 1.1;<br>Figure 6, 1.1	The longitudinal activity contrast and mortality outcomes were sufficiently comparable with the corresponding maintained/continued-activity strata.
**C30**<br>**Biscaglia et al. (2020)**<br>*Prospective registry cohort*	Habitual physical-activity association	Single self-reported baseline item: sedentary; light activity most weeks; vigorous activity for ≥20 min once/twice weekly; or vigorous activity ≥3 times/week. The displayed contrast is vigorous once/twice weekly versus light.	Core 3-point MACE	**Displayed separately; not pooled**<br>Figure 7, 1.1	Unique activity-intensity contrast. Although the outcome was core MACE, the exposure contrast was not equivalent to longitudinal change, target achievement, walking, or pre-admission activity.

Report and study design	Evidence question / exposure class	Assessment instrument or intervention protocol; scale, metric, and operational contrast	Eligible outcome(s)	Synthesis contribution and location	Reason for non-pooling or handling decision
**C31**<br>**Lahtinen et al. (2018)**<br>*Prospective cohort*	Longitudinal physical-activity association	Validated modified Saltin-Grimby single-item scale at baseline and 2 years: inactive; irregular light activity; moderate activity 2–3 times/week; or moderate/high activity >3 times/week, with each session 30 min. Longitudinal active versus inactive patterns.	Cardiovascular mortality	**Pooled (estimand-specific)**<br>Figure 6, 1.1	The longitudinal cardiovascular-mortality contrast was sufficiently comparable with the maintained/continued-activity stratum.
**C32**<br>**Jin et al. (2022)**<br>*Registry cohort*	Habitual physical-activity association	Self-reported weekly duration of light, moderate, and vigorous activity converted to MET-min/week. Inactive 0, insufficient 1–<500, target 500–<1000, and high ≥1000; target versus inactivity.	All-cause mortality; MACE	**Pooled for all-cause mortality; MACE displayed separately**<br>Figure 5, 1.2;<br>Figure 7, 1.3	The all-cause mortality contrast entered the target-versus-inactivity stratum. The MACE estimate was displayed separately because the outcome and exposure contrast were not equivalent to the other MACE studies.
**C33**<br>**Moholdt et al. (2017)**<br>*Prospective cohort*	Habitual physical-activity association	Self-reported HUNT questions on leisure-time activity frequency, duration, and intensity at each wave; classified as inactive, below recommendations, meeting recommendations, or exceeding recommendations; meeting target versus inactivity.	All-cause and cardiovascular mortality	**Pooled (estimand-specific)**<br>Figure 5, 1.2;<br>Figure 6, 1.2	The target-versus-inactivity contrast and mortality outcomes were sufficiently comparable with the corresponding strata.
**C09†**<br>**Stewart et al. (2017)**<br>*Prospective trial-cohort analysis*	Habitual exercise-volume association	Self-reported questionnaire based on IPAQ; weekly hours of mild, moderate, and vigorous activity assigned 2, 4, and 8 METs, respectively. Total MET-h/week analyzed per doubling of habitual exercise volume.	All-cause and cardiovascular mortality	**Displayed separately; not pooled**<br>Figure 5, 1.5;<br>Figure 6, 1.4	Continuous dose contrast was not equivalent to categorical activity contrasts. The report shared the STABILITY parent cohort with Stewart et al. (2016), which addressed Mediterranean diet adherence.

Report and study design	Evidence question / exposure class	Assessment instrument or intervention protocol; scale, metric, and operational contrast	Eligible outcome(s)	Synthesis contribution and location	Reason for non-pooling or handling decision
**C34**<br>**Bouisset et al. (2020)**<br>*Prospective cohort*	Habitual physical-activity association	Interviewer-administered validated MOSPA questionnaire covering occupational, commuting, household, and leisure activity in the previous year; total MET-h/week; tertiles 0–9.9, 10–39.9, and ≥40; highest versus lowest.	All-cause mortality	**Pooled (estimand-specific)**<br>Figure 5, 1.3	The categorical all-cause mortality contrast was sufficiently comparable with Ek and Li and was pooled only within that estimand-specific stratum.

Abbreviations: ACS, acute coronary syndrome; aMED, alternate Mediterranean Diet score; CAD, coronary artery disease; CHD, coronary heart disease; CR, cardiac rehabilitation; CVD, cardiovascular disease; FFQ, food-frequency questionnaire; HR, hazard ratio; IPAQ, International Physical Activity Questionnaire; IQR, interquartile range; LTPA, leisure-time physical activity; MACE, major adverse cardiovascular events; MACCE, major adverse cardiac and cerebrovascular events; MDS, Mediterranean Diet Score; MEDLIFE, Mediterranean lifestyle; MET, metabolic equivalent of task; MI, myocardial infarction; MINOCA, myocardial infarction with non-obstructive coronary arteries; MVPA, moderate-to-vigorous physical activity; NHANES, National Health and Nutrition Examination Survey; NHIRD, National Health Insurance Research Database; NRSI, non-randomized study of an intervention; OMT, optimal medical therapy; PA, physical activity; PCI, percutaneous coronary intervention; RCT, randomized controlled trial; RR, risk ratio; SD, standard deviation; STEMI, ST-segment elevation myocardial infarction.

Supplementary Table S5.

Timing of Mediterranean diet or lifestyle assessment relative to coronary heart disease and consideration of secondary-prevention treatment

Assessment timing refers to the dietary or lifestyle measure used in the outcome analysis. None of the included reports assessed lifetime Mediterranean diet adherence.

Study	Evidence type	Timing of dietary/lifestyle assessment relative to CHD	Secondary-prevention treatment context
Delgado-Lista et al. (2022)	RCT	Mediterranean diet assigned after established CHD; median follow-up was 7.0 years.	Baseline pharmacotherapy recorded; randomization and intention-to-treat analysis used.
Cangemi et al. (2023)	Observational adherence	Assessed at hospital admission/enrolment using a 14-item questionnaire with dietitian assistance; exact dietary look-back period not reported.	Adjusted for age, sex, BMI, DASI, obstructive CAD, heart failure, and type 2 diabetes; pharmacotherapy not comprehensively adjusted.
Shikany et al. (2018)	Observational adherence	Baseline Block 98 food-frequency questionnaire among participants with CHD; assessed usual dietary intake.	Broad adjustment for demographic, socioeconomic, behavioural, adiposity, blood-pressure, and cardiometabolic factors; medication sensitivity analysis performed.
Díaz-Gutiérrez et al. (2026)	Observational joint-lifestyle	MEDLIFE questionnaire administered once at baseline after enrolment with ischemic heart disease and/or atrial fibrillation.	Adjusted for prior CVD, cardiac rehabilitation, cardiovascular risk-factor count, cardiovascular-drug count, LDL-C, and HbA1c.
Lau et al. (2015)	Observational adherence	Baseline validated food-frequency questionnaire in stable CAD; dietary pattern and cardiovascular medications stable for at least 3 months.	Adjusted for mean systolic blood pressure, demographic and clinical risk factors, antithrombotic agents, statins, and antihypertensive medications.

Study	Evidence type	Timing of dietary/lifestyle assessment relative to CHD	Secondary-prevention treatment context
Kouvari et al. (2017)	Observational adherence	Food-frequency questionnaire at hospitalization for first-diagnosed ACS; exact pre-event recall window not reported.	First-diagnosed ACS reduced prior-treatment confounding; adjusted for demographic/clinical risk factors, physical activity, C-reactive protein, and left ventricular ejection fraction; medication adjustment incomplete.
Magnoni et al. (2020)	Observational pre-event diet	Pre-STEMI dietary habits assessed retrospectively by food-frequency questionnaire at admission; first STEMI without previous CHD.	Acute treatment and cardiovascular risk factors recorded; background pharmacotherapy not comprehensively adjusted.
Liang et al. (2022)	NHANES observational adherence	Diet and aMED score assessed at survey baseline in participants reporting previous CHD or stroke; interval since diagnosis unavailable.	Models adjusted for age, sex, BMI, race, hypertension, and energy intake; medications and CHD severity were not included.
Pan et al. (2025)	NHANES observational adherence	Baseline 24-hour dietary recalls in participants reporting previous CVD; interval since diagnosis unavailable.	Models adjusted for demographic, socioeconomic, anthropometric, and lifestyle factors and energy intake; medications and disease severity were not included.
Stewart et al. (2016)	Observational adherence (STABILITY)	Lifestyle food-frequency questionnaire administered once at baseline among patients with established stable CHD.	Adjusted for trial assignment, demographic factors, smoking, CHD severity, cardiovascular risk factors, physical activity, region, income, education, and the alternative dietary score.

Abbreviations: ACS, acute coronary syndrome; aMED, alternative Mediterranean diet score; BMI, body mass index; CAD, coronary artery disease; CHD, coronary heart disease; CVD, cardiovascular disease; FFQ, food-frequency questionnaire; HbA1c, glycated hemoglobin; LDL-C, low-density lipoprotein cholesterol; MEDLIFE, Mediterranean lifestyle index; NHANES, National Health and Nutrition Examination Survey; STEMI, ST-segment elevation myocardial infarction.
