# Supplementary material for: Association of the Mediterranean Diet, Physical Activity and Related Lifestyle Factors on the Mortality and Major Adverse Cardiovascular Events in Patients with Coronary Heart Disease: A Systematic Review and Meta-Analysis
Source: Nutrients. 2026 Aug 7;18(16):2589. doi: 10.3390/nu18162589 (PMC13516061; doi:10.3390/nu18162589)
Supplement: Supplementary file 1 [file nutrients-18-02589-s001.zip › Supplementary Material File S2.pdf]

Supplementary Material File S2

Source Source	Final search date	Records retrieved	Notes
PubMed (National Library of Medicine)	June 15, 2026	1,271	Count reported in the PRISMA flow diagram
MEDLINE via Ovid	June 15, 2026	1,707	Count reported in the PRISMA flow diagram
Scopus (Elsevier)	June 15, 2026	4,052	Count reported in the PRISMA flow diagram
Embase (Embase.com)	June 15, 2026	4,148	Count reported in the PRISMA flow diagram
Cochrane Library (CENTRAL)	June 15, 2026	586	Count reported in the PRISMA flow diagram
**Total before deduplication**	/	**11,764**	**6,231 duplicates removed; 5,533 unique records screened**

PubMed

**Database/platform**	PubMed (National Library of Medicine)	**Records retrieved**	1,271
**Final search date**	June 15, 2026	**Date restriction**	January 1, 2015 to June 15, 2026
**Language restriction**	English or Chinese	**Other filters**	No study-design or other methodological filter
**Controlled vocabulary**	MeSH terms in the archived strategy: "Diet, Mediterranean"[Mesh] and "Mortality"[MeSH].	**Free-text fields**	Title/abstract terms ([tiab]/[Title/Abstract]) and [All Fields].
**Field tags**	[Mesh], [MeSH], [tiab], [Title/Abstract], and [All Fields]	**Limit application**	Date and language limits were applied through the database interface when not embedded in the archived query.

Search line	Exact strategy as recorded
**#1**	("Diet, Mediterranean"[Mesh] OR "Mediterranean Diet"[tiab] OR "Mediterranean dietary pattern"[tiab] OR "mediterranean lifestyle"[tiab] OR "mediterranean diet index"[tiab] OR "mediterranean style diet"[tiab] OR "mediterranean diet score"[tiab])
**#2**	("exercise"[All Fields] OR "training"[All Fields] OR "exercise training"[All Fields] OR "physical activity"[All Fields] OR "athletes"[All Fields] OR "aerobic exercise"[All Fields] OR "aerobic training"[All Fields] OR "endurance exercise"[All Fields] OR "endurance training"[All Fields] OR "resistance exercise"[All Fields] OR "resistance training"[All Fields] OR "strength exercise"[All Fields] OR "strength training"[All Fields] OR "combined exercise"[All Fields] OR "combined training"[All Fields] OR "concurrent exercise"[All Fields] OR "concurrent training"[All Fields] OR "sports"[All Fields] OR "exercise therapy"[All Fields] OR "lifestyle intervention"[All Fields] OR "anaerobic training"[All Fields])
**#3**	((Coronary Diseases[Title/Abstract]) OR (Disease*, Coronary[Title/Abstract]) OR (Coronary Heart Disease*[Title/Abstract]) OR (Disease*, Coronary Heart[Title/Abstract]) OR (Heart Disease*, Coronary[Title/Abstract]) OR (Ischemia*, Myocardial[Title/Abstract]) OR (Myocardial Ischemia*[Title/Abstract]) OR (Ischemic Heart Disease*[Title/Abstract]) OR (Heart Disease*, Ischemic[Title/Abstract]) OR (Disease*, Ischemic Heart[Title/Abstract]) OR (Artery Disease*, Coronary[Title/Abstract]) OR (Coronary Artery Disease*[Title/Abstract]) OR (Left Main Coronary Artery Disease[Title/Abstract]) OR (Left Main Disease*[Title/Abstract]) OR (Left Main Coronary Disease[Title/Abstract]) OR (Coronary Arterioscleros*[Title/Abstract]) OR (Coronary Atheroscleros*[Title/Abstract]) OR (Acute Coronary Syndrome*[Title/Abstract]) OR (Coronary Syndrome*, Acute[Title/Abstract]) OR (Syndrome*, Acute Coronary[Title/Abstract]) OR (Coronary Intervention*, Percutaneous[Title/Abstract]) OR (Intervention*, Percutaneous Coronary[Title/Abstract]) OR (Percutaneous Coronary Interventions[Title/Abstract]) OR (Percutaneous Coronary Revascularization*[Title/Abstract]) OR (Coronary Revascularization*, Percutaneous[Title/Abstract]) OR (Revascularization*, Percutaneous Coronary[Title/Abstract]) OR (Percutaneous Transluminal Coronary Angioplast*[Title/Abstract]) OR (Artery Bypass*, Coronary[Title/Abstract]) OR (Bypass*, Coronary Artery[Title/Abstract]) OR (Coronary Artery Bypasses[Title/Abstract]) OR (Coronary Artery Bypass Surgery[Title/Abstract]) OR (Coronary Artery Bypass Grafting[Title/Abstract]) OR (Aortocoronary Bypass*[Title/Abstract]) OR (Bypass*, Aortocoronary[Title/Abstract]) OR (Bypass Surgery, Coronary Artery[Title/Abstract]) OR (Myocardial Revascularizations[Title/Abstract]) OR (Revascularization*, Myocardial[Title/Abstract]) OR (Internal Mammary Artery Implantation[Title/Abstract]))
**#4**	("Mortality"[MeSH] OR mortality[tiab] OR death[tiab] OR deaths[tiab] OR "Cardiovascular mortality"[tiab] OR "All-cause mortality"[tiab] OR "All cause mortality"[tiab] OR "Major adverse cardiovascular event"[tiab] OR "Major adverse cardiovascular events"[tiab] OR "Major adverse cardiac event"[tiab] OR "Major adverse cardiac events"[tiab] OR "Major

Search line	Exact strategy as recorded
	adverse cardiovascular and cerebrovascular event"[tiab] OR "Major adverse cardiovascular and cerebrovascular events"[tiab] OR MACE[tiab] OR MACCE[tiab] OR "cardiovascular event"[tiab] OR "cardiovascular events"[tiab] OR "cardiac event"[tiab] OR "cardiac events"[tiab] OR "coronary event"[tiab] OR "coronary events"[tiab] OR "cardiovascular outcome"[tiab] OR "cardiovascular outcomes"[tiab] OR "cardiac outcome"[tiab] OR "cardiac outcomes"[tiab] OR "myocardial infarction"[tiab] OR stroke[tiab])
**Mediterranean diet and Coronary heart disease**	#1 AND #3 AND #4
**Exercise and Coronary heart disease**	#2 AND #3 AND #4
**Mediterranean diet and Exercise and Coronary heart disease**	#1 AND #2 AND #3 AND #4

MEDLINE

**Database/platform**	MEDLINE via Ovid	**Records retrieved**	1,707
**Final search date**	June 15, 2026	**Date restriction**	January 1, 2015 to June 15, 2026
**Language restriction**	English or Chinese	**Other filters**	No study-design or other methodological filter
**Controlled vocabulary**	Exploded MeSH headings: Mediterranean Diet, Exercise, Coronary Disease, Mortality, Myocardial Infarction, Stroke, Myocardial Revascularization, and Hospitalization.	**Free-text fields**	Free-text terms searched in title and abstract fields.
**Field tags**	exp [MeSH heading]/ and .ti,ab.	**Limit application**	Date and language limits were applied through the database interface when not embedded in the archived query.

**Search line**	**Exact strategy as recorded**
**#1**	exp Mediterranean Diet/ OR (mediterranean diet OR mediterranean lifestyle OR mediterranean style diet OR mediterranean diet score OR mediterranean diet index).ti,ab.
**#2**	exp Exercise/ OR (exercise OR physical activity OR sports OR training OR exercise training OR aerobic exercise OR aerobic training OR endurance exercise OR endurance training OR resistance exercise OR resistance training OR strength exercise OR strength training OR combined exercise OR combined training OR concurrent exercise OR concurrent training OR exercise therapy OR lifestyle intervention OR anaerobic training).ti,ab.
**#3**	exp Coronary Disease/ OR (Coronary Diseases OR Coronary Heart Disease OR Ischemic Heart Disease OR Coronary Artery Disease OR Left Main Coronary Artery Disease OR Coronary Arterioscleros* OR Coronary Atheroscleros* OR Acute Coronary Syndrome OR Percutaneous Coronary Intervention* OR Percutaneous Coronary Revascularization OR Percutaneous Transluminal Coronary Angioplast* OR Coronary Artery Bypass* OR Coronary Artery Bypass Grafting OR Myocardial Revascularization).ti,ab.
**#4**	exp Mortality/ OR exp Myocardial Infarction/ OR exp Stroke/ OR exp Myocardial Revascularization/ OR exp Hospitalization/ OR (mortality OR death OR deaths OR cardiovascular mortality OR all-cause mortality OR all cause mortality OR major adverse cardiovascular event* OR major adverse cardiac event* OR major adverse cardiovascular and cerebrovascular event* OR MACE OR MACCE OR cardiovascular event* OR cardiac event* OR coronary event* OR cardiovascular outcome* OR cardiac outcome* OR myocardial infarction OR stroke).ti,ab.
**Mediterranean diet and Coronary heart disease**	#1 AND #3 AND #4
**Exercise and Coronary heart disease**	#2 AND #3 AND #4
**Mediterranean diet and Exercise and Coronary heart disease**	#1 AND #2 AND #3 AND #4

Scopus

**Database/platform**	Scopus (Elsevier)	**Records retrieved**	4,052
**Final search date**	June 15, 2026	**Date restriction**	January 1, 2015 to June 15, 2026
**Language restriction**	English or Chinese	**Other filters**	No study-design or other methodological filter
**Controlled vocabulary**	Not applicable; Scopus does not provide a controlled-vocabulary thesaurus.	**Free-text fields**	Free-text terms searched in title, abstract, and author-keyword fields.
**Field tags**	TITLE-ABS-KEY	**Limit application**	Date and language limits were applied through the database interface when not embedded in the archived query.

**Search line**	**Exact strategy as recorded**
**Mediterranean diet and Coronary heart disease**	TITLE-ABS-KEY ("mediterranean diet" OR "mediterranean lifestyle" OR "mediterranean diet index" OR "mediterranean style diet" OR "mediterranean diet score") AND TITLE-ABS-KEY ("Coronary Diseases" OR "Coronary Heart Disease" OR "Ischemic Heart Disease" OR "Coronary Artery Disease" OR "Left Main Coronary Artery Disease" OR "Coronary Arterioscleros*" OR "Coronary Atheroscleros*" OR "Acute Coronary Syndrome" OR "Percutaneous Coronary Interventions" OR "Percutaneous Coronary Revascularization" OR "Percutaneous Transluminal Coronary Angioplast*" OR "Coronary Artery Bypasses" OR "Coronary Artery Bypass Grafting" OR "Myocardial Revascularization") AND TITLE-ABS-KEY ("mortality" OR "death" OR "deaths" OR "cardiovascular mortality" OR "all-cause mortality" OR "all cause mortality" OR "major adverse cardiovascular event*" OR "major adverse cardiac event*" OR "major adverse cardiovascular and cerebrovascular event*" OR MACE OR MACCE OR "cardiovascular event*" OR "cardiac event*" OR "coronary event*" OR "cardiovascular outcome*" OR "cardiac outcome*" OR "myocardial infarction" OR "stroke")
**Exercise and Coronary heart disease**	TITLE-ABS-KEY ("exercise" OR "physical activity" OR "sports" OR "training" OR "exercise training" OR "aerobic exercise" OR "aerobic training" OR "endurance exercise" OR "endurance training" OR "resistance exercise" OR "resistance training" OR "strength exercise" OR "strength training" OR "combined exercise" OR "combined training" OR "concurrent exercise" OR "concurrent training" OR "exercise therapy" OR "lifestyle intervention" OR "anaerobic training") AND TITLE-ABS-KEY ("Coronary Diseases" OR "Coronary Heart Disease" OR "Ischemic Heart Disease" OR "Coronary Artery Disease" OR "Left Main Coronary Artery Disease" OR "Coronary Arterioscleros*" OR "Coronary Atheroscleros*" OR "Acute Coronary Syndrome" OR "Percutaneous Coronary Interventions" OR "Percutaneous Coronary Revascularization" OR "Percutaneous Transluminal Coronary Angioplast*" OR "Coronary Artery Bypasses" OR "Coronary Artery Bypass Grafting" OR "Myocardial Revascularization") AND TITLE-ABS-KEY ("mortality" OR "death" OR "deaths" OR "cardiovascular mortality" OR "all-cause mortality" OR "all cause mortality" OR "major adverse cardiovascular event*" OR "major adverse cardiac event*" OR "major adverse cardiovascular and cerebrovascular event*" OR MACE OR MACCE OR "cardiovascular event*" OR "cardiac event*" OR "coronary event*" OR "cardiovascular outcome*" OR "cardiac outcome*" OR "myocardial infarction" OR "stroke")
**Mediterranean diet and Exercise and Coronary heart disease**	TITLE-ABS-KEY ("mediterranean diet" OR "mediterranean lifestyle" OR "mediterranean diet index" OR "mediterranean style diet" OR "mediterranean diet score") AND TITLE-ABS-KEY ("exercise" OR "physical activity" OR "sports" OR "training" OR "exercise training" OR "aerobic exercise" OR "aerobic training" OR "endurance exercise" OR "endurance training" OR "resistance exercise" OR "resistance training" OR "strength exercise" OR "strength training" OR "combined exercise" OR "combined training" OR "concurrent exercise" OR "concurrent training" OR "exercise therapy" OR "lifestyle intervention" OR "anaerobic training") AND TITLE-ABS-KEY ("Coronary Diseases" OR "Coronary Heart Disease" OR "Ischemic Heart Disease" OR "Coronary Artery Disease" OR "Left Main Coronary Artery Disease" OR "Coronary Arterioscleros*" OR "Coronary Atheroscleros*" OR "Acute Coronary Syndrome" OR "Percutaneous Coronary Interventions" OR "Percutaneous Coronary Revascularization" OR "Percutaneous Transluminal Coronary Angioplast*" OR "Coronary Artery Bypasses" OR "Coronary Artery Bypass Grafting" OR "Myocardial Revascularization") AND TITLE-ABS-KEY ("mortality" OR "death" OR "deaths" OR "cardiovascular mortality" OR "all-cause mortality" OR "all cause mortality" OR "major adverse cardiovascular event*" OR "major adverse cardiac event*" OR "major adverse cardiovascular and cerebrovascular event*" OR MACE OR MACCE OR "cardiovascular event*" OR "cardiac event*" OR "coronary event*" OR "cardiovascular outcome*" OR "cardiac outcome*" OR "myocardial infarction" OR "stroke")

Embase

Database/platform	Embase (Embase.com)	Records retrieved	4,148
Final search date	June 15, 2026	Date restriction	January 1, 2015 to June 15, 2026
Language restriction	English or Chinese	Other filters	No study-design or other methodological filter
Controlled vocabulary	No Emtree headings were present in the archived strategy; the exact search was based on free-text terms.	Free-text fields	Free-text terms searched in title and abstract fields.
Field tags	:ti,ab	Limit application	Date and language limits were applied through the database interface when not embedded in the archived query.

Search line	Exact strategy as recorded
Mediterranean diet and Coronary heart disease	('mediterranean diet' OR 'mediterranean lifestyle' OR 'mediterranean style diet' OR 'mediterranean diet score' OR 'mediterranean diet index'):ti,ab AND ('Coronary Diseases' OR 'Coronary Heart Disease' OR 'Ischemic Heart Disease' OR 'Coronary Artery Disease' OR 'Left Main Coronary Artery Disease' OR 'Coronary Arterioscleros*' OR 'Coronary Atheroscleros*' OR 'Acute Coronary Syndrome' OR 'Percutaneous Coronary Interventions' OR 'Percutaneous Coronary Revascularization' OR 'Percutaneous Transluminal Coronary Angioplast*' OR 'Coronary Artery Bypasses' OR 'Coronary Artery Bypass Grafting' OR 'Myocardial Revascularization'):ti,ab AND ('mortality' OR 'death' OR 'deaths' OR 'cardiovascular mortality' OR 'all-cause mortality' OR 'all cause mortality' OR 'major adverse cardiovascular event*' OR 'major adverse cardiac event*' OR 'major adverse cardiovascular and cerebrovascular event*' OR MACE OR MACCE OR 'cardiovascular event*' OR 'cardiac event*' OR 'coronary event*' OR 'cardiovascular outcome*' OR 'cardiac outcome*' OR 'myocardial infarction' OR 'stroke'):ti,ab
Exercise and Coronary heart disease	('exercise' OR 'physical activity' OR 'sports' OR 'training' OR 'exercise training' OR 'aerobic exercise' OR 'aerobic training' OR 'endurance exercise' OR 'endurance training' OR 'resistance exercise' OR 'resistance training' OR 'strength exercise' OR 'strength training' OR 'combined exercise' OR 'combined training' OR 'concurrent exercise' OR 'concurrent training' OR 'exercise therapy' OR 'lifestyle intervention' OR 'anaerobic training'):ti,ab AND ('Coronary Diseases' OR 'Coronary Heart Disease' OR 'Ischemic Heart Disease' OR 'Coronary Artery Disease' OR 'Left Main Coronary Artery Disease' OR 'Coronary Arterioscleros*' OR 'Coronary Atheroscleros*' OR 'Acute Coronary Syndrome' OR 'Percutaneous Coronary Interventions' OR 'Percutaneous Coronary Revascularization' OR 'Percutaneous Transluminal Coronary Angioplast*' OR 'Coronary Artery Bypasses' OR 'Coronary Artery Bypass Grafting' OR 'Myocardial Revascularization'):ti,ab AND ('mortality' OR 'death' OR 'deaths' OR 'cardiovascular mortality' OR 'all-cause mortality' OR 'all cause mortality' OR 'major adverse cardiovascular event*' OR 'major adverse cardiac event*' OR 'major adverse cardiovascular and cerebrovascular event*' OR MACE OR MACCE OR 'cardiovascular event*' OR 'cardiac event*' OR 'coronary event*' OR 'cardiovascular outcome*' OR 'cardiac outcome*' OR 'myocardial infarction' OR 'stroke'):ti,ab
Mediterranean diet and Exercise and Coronary heart disease	('mediterranean diet' OR 'mediterranean lifestyle' OR 'mediterranean style diet' OR 'mediterranean diet score' OR 'mediterranean diet index'):ti,ab AND ('exercise' OR 'physical activity' OR 'sports' OR 'training' OR 'exercise training' OR 'aerobic exercise' OR 'aerobic training' OR 'endurance exercise' OR 'endurance training' OR 'resistance exercise' OR 'resistance training' OR 'strength exercise' OR 'strength training' OR 'combined exercise' OR 'combined training' OR 'concurrent exercise' OR 'concurrent training' OR 'exercise therapy' OR 'lifestyle intervention' OR 'anaerobic training'):ti,ab AND ('Coronary Diseases' OR 'Coronary Heart Disease' OR 'Ischemic Heart Disease' OR 'Coronary Artery Disease' OR 'Left Main Coronary Artery Disease' OR 'Coronary Arterioscleros*' OR 'Coronary Atheroscleros*' OR 'Acute Coronary Syndrome' OR 'Percutaneous Coronary Interventions' OR 'Percutaneous Coronary Revascularization' OR 'Percutaneous Transluminal Coronary Angioplast*' OR 'Coronary Artery Bypasses' OR 'Coronary Artery Bypass Grafting' OR 'Myocardial Revascularization'):ti,ab AND ('mortality' OR 'death' OR 'deaths' OR 'cardiovascular mortality' OR 'all-cause mortality' OR 'all cause mortality' OR 'major adverse cardiovascular event*' OR 'major adverse cardiac event*' OR 'major adverse cardiovascular and cerebrovascular event*' OR MACE OR MACCE OR 'cardiovascular event*' OR 'cardiac event*' OR 'coronary event*' OR 'cardiovascular outcome*' OR 'cardiac outcome*' OR 'myocardial infarction' OR 'stroke'):ti,ab

Cochrane Library/CENTRAL

**Database/platform**	Cochrane Library (CENTRAL)	**Records retrieved**	586
**Final search date**	June 15, 2026	**Date restriction**	January 1, 2015 to June 15, 2026
**Language restriction**	English or Chinese	**Other filters**	No study-design or other methodological filter
**Controlled vocabulary**	No MeSH descriptor was present in the archived strategy; the exact search was based on free-text terms.	**Free-text fields**	Free-text terms searched in All Text.
**Field tags**	in All Text	**Limit application**	Date and language limits were applied through the database interface when not embedded in the archived query.

Search line	Exact strategy as recorded
**Mediterranean diet and Coronary heart disease**	("mediterranean diet" OR "mediterranean lifestyle" OR "mediterranean style diet" OR "mediterranean diet score" OR "mediterranean diet index") in All Text AND ("Coronary Diseases" OR "Coronary Heart Disease" OR "Ischemic Heart Disease" OR "Coronary Artery Disease" OR "Left Main Coronary Artery Disease" OR "Coronary Arterioscleros*" OR "Coronary Atheroscleros*" OR "Acute Coronary Syndrome" OR "Percutaneous Coronary Interventions" OR "Percutaneous Coronary Revascularization" OR "Percutaneous Transluminal Coronary Angioplast*" OR "Coronary Artery Bypasses" OR "Coronary Artery Bypass Grafting" OR "Myocardial Revascularization") in All Text AND ("mortality" OR "death" OR "deaths" OR "cardiovascular mortality" OR "all-cause mortality" OR "all cause mortality" OR "major adverse cardiovascular event*" OR "major adverse cardiac event*" OR "major adverse cardiovascular and cerebrovascular event*" OR MACE OR MACCE OR "cardiovascular event*" OR "cardiac event*" OR "coronary event*" OR "cardiovascular outcome*" OR "cardiac outcome*" OR "myocardial infarction" OR "stroke") in All Text
**Exercise and Coronary heart disease**	("exercise" OR "physical activity" OR "sports" OR "training" OR "exercise training" OR "aerobic exercise" OR "aerobic training" OR "endurance exercise" OR "endurance training" OR "resistance exercise" OR "resistance training" OR "strength exercise" OR "strength training" OR "combined exercise" OR "combined training" OR "concurrent exercise" OR "concurrent training" OR "exercise therapy" OR "lifestyle intervention" OR "anaerobic training") in All Text AND ("Coronary Diseases" OR "Coronary Heart Disease" OR "Ischemic Heart Disease" OR "Coronary Artery Disease" OR "Left Main Coronary Artery Disease" OR "Coronary Arterioscleros*" OR "Coronary Atheroscleros*" OR "Acute Coronary Syndrome" OR "Percutaneous Coronary Interventions" OR "Percutaneous Coronary Revascularization" OR "Percutaneous Transluminal Coronary Angioplast*" OR "Coronary Artery Bypasses" OR "Coronary Artery Bypass Grafting" OR "Myocardial Revascularization") in All Text AND ("mortality" OR "death" OR "deaths" OR "cardiovascular mortality" OR "all-cause mortality" OR "all cause mortality" OR "major adverse cardiovascular event*" OR "major adverse cardiac event*" OR "major adverse cardiovascular and cerebrovascular event*" OR MACE OR MACCE OR "cardiovascular event*" OR "cardiac event*" OR "coronary event*" OR "cardiovascular outcome*" OR "cardiac outcome*" OR "myocardial infarction" OR "stroke") in All Text
**Mediterranean diet and Exercise and Coronary heart disease**	("mediterranean diet" OR "mediterranean lifestyle" OR "mediterranean style diet" OR "mediterranean diet score" OR "mediterranean diet index") in All Text AND ("exercise" OR "physical activity" OR "sports" OR "training" OR "exercise training" OR "aerobic exercise" OR "aerobic training" OR "endurance exercise" OR "endurance training" OR "resistance exercise" OR "resistance training" OR "strength exercise" OR "strength training" OR "combined exercise" OR "combined training" OR "concurrent exercise" OR "concurrent training" OR "exercise therapy" OR "lifestyle intervention" OR "anaerobic training") in All Text AND ("Coronary Diseases" OR "Coronary Heart Disease" OR "Ischemic Heart Disease" OR "Coronary Artery Disease" OR "Left Main Coronary Artery Disease" OR "Coronary Arterioscleros*" OR "Coronary Atheroscleros*" OR "Acute Coronary Syndrome" OR "Percutaneous Coronary Interventions" OR "Percutaneous Coronary Revascularization" OR "Percutaneous Transluminal Coronary Angioplast*" OR "Coronary Artery Bypasses" OR "Coronary Artery Bypass Grafting" OR "Myocardial Revascularization") in All Text AND ("mortality" OR "death" OR "deaths" OR "cardiovascular mortality" OR "all-cause mortality" OR "all cause mortality" OR "major adverse cardiovascular event*" OR "major adverse cardiac event*" OR "major adverse cardiovascular and cerebrovascular event*" OR MACE OR MACCE OR "cardiovascular event*" OR "cardiac event*" OR "coronary event*" OR "cardiovascular outcome*" OR "cardiac outcome*" OR "myocardial infarction" OR "stroke") in All Text

Abbreviations: CENTRAL, Cochrane Central Register of Controlled Trials; MACE, major adverse cardiovascular events; MACCE, major adverse cardiac and cerebrovascular events; MeSH, Medical Subject Headings.
